# One Step Nucleic Acid Amplification (OSNA) Lysate Samples Are Suitable to Establish a Transcriptional Metastatic Signature in Patients with Early Stage Hormone Receptors-Positive Breast Cancer

**DOI:** 10.3390/cancers14235855

**Published:** 2022-11-28

**Authors:** Inês Gante, Joana Martins Ribeiro, João Mendes, Ana Gomes, Vânia Almeida, Frederico Soares Regateiro, Francisco Caramelo, Henriqueta Coimbra Silva, Margarida Figueiredo-Dias

**Affiliations:** 1 Gynecology Department, Coimbra Hospital and Universitary Centre (CHUC), 3004-561 Coimbra, Portugal; 2 University of Coimbra, Gynecology University Clinic, Faculty of Medicine, 3000-548 Coimbra, Portugal; 3 Coimbra Institute for Clinical and Biomedical Research (iCBR) Area of Environment, Genetics and Oncobiology (CIMAGO), Faculty of Medicine, University of Coimbra, 3000-548 Coimbra, Portugal; 4 Laboratory of Sequencing and Functional Genomics of UCGenomics, Faculty of Medicine, University of Coimbra, 3000-548 Coimbra, Portugal; 5 Department of Pathology, Coimbra Hospital and Universitary Centre (CHUC), 3004-561 Coimbra, Portugal; 6 Institute of Anatomical and Molecular Pathology, Faculty of Medicine, University of Coimbra, 3000-548 Coimbra, Portugal; 7 Allergy and Clinical Immunology Unit, Coimbra Hospital and Universitary Centre (CHUC), 3004-561 Coimbra, Portugal; 8 Institute of Immunology, Faculty of Medicine, University of Coimbra, 3000-548 Coimbra, Portugal; 9 Laboratory of Biostatistics and Medical Informatics (LBIM), Faculty of Medicine, University of Coimbra, 3000-548 Coimbra, Portugal; 10 Center for Innovative Biomedicine and Biotechnology (CIBB), Faculty of Medicine, University of Coimbra, 3000-548 Coimbra, Portugal

**Keywords:** breast neoplasms, lymph nodes, OSNA, metastases, biomarkers, immune system, immunotherapy

## Abstract

**Simple Summary:**

The One Step Nucleic Acid Amplification (OSNA) is a recent technique for sentinel lymph nodes staging in breast cancer (BC). After OSNA assay, instead of being discarded, the residual OSNA lysate can also be used for gene expression studies, being the potentially ideal sample to search for new biomarkers. The aim of our study was to identify biomarkers related to tumor-microenvironment interplay in OSNA lysate of sentinel lymph nodes in women with early stage hormone receptors-positive BC. We identified 11 upregulated genes in metastatic lymph nodes. These genes codify proteins mainly involved in cancer aggressiveness and with impact in immune response. Thus, these findings support that OSNA lysate transcriptomic analysis may identify biomarkers potentially useful in the future for prognosis stratification and therapy selection. As OSNA assay is being implemented for sentinel lymph nodes staging in other cancers, this approach could also have a wider utility.

**Abstract:**

The One Step Nucleic Acid Amplification (OSNA) is being adopted worldwide for sentinel lymph nodes (SLNs) staging in breast cancer (BC). As major disadvantage, OSNA precludes prognostic information based on structural evaluation of SLNs. Our aim is to identify biomarkers related to tumor-microenvironment interplay exploring gene expression data from the OSNA remaining lysate. This study included 32 patients with early stage hormone receptors-positive BC. Remaining OSNA lysates were prepared for targeted RNA-sequencing analysis. Identification of differentially expressed genes (DEGs) was performed by DESeq2 in R and data analysis in STATA. The results show that, in metastatic SLNs, several genes were upregulated: *KRT7*, *VTCN1*, *CD44*, *GATA3*, *ALOX15B*, *RORC*, *NECTIN2*, *LRG1*, *CD276*, *FOXM1* and *IGF1R*. Hierarchical clustering analysis revealed three different clusters. The identified DEGs codify proteins mainly involved in cancer aggressiveness and with impact in immune response. The overexpression of the immune suppressive genes *VTCN1* and *CD276* may explain that no direct evidence of activation of immune response in metastatic SLNs was found. We show that OSNA results may be improved incorporating microenvironment-related biomarkers that may be useful in the future for prognosis stratification and immunotherapy selection. As OSNA assay is being implemented for SLNs staging in other cancers, this approach could also have a wider utility.

## 1. Introduction

Lymph nodes (LNs) are the main doorway for tumor cell metastases and its evaluation is a major prognostic factor. Two-thirds of breast cancer (BC) patients diagnosed with LNs metastases will develop distant metastases and 73% of these women will be dead within 5 years after diagnosis [1,2]. SLN biopsy is the standard approach for loco-regional staging in patients with clinically T1-T2 invasive BC presenting with a clinically negative axilla [3,4]. Patients with negative SLNs require no further axillary surgery [5,6]. In patients with 1 or 2 metastatic SLNs who meet the criteria of ACOSOG Z0011 or AMAROS trials, completion of axillary LN dissection (ALND) is not necessary if irradiation and systemic adjuvant therapy are planned [3,7,8,9,10]. However, currently, for Luminal [estrogen receptors (ER) positive] BC with metastatic LNs, it is recommended an extension of endocrine therapy towards a duration of 10 years based on persistent risks of recurrence among such patients [5,6,11].

Conventional intraoperative histological examinations of SLNs frozen sections are associated with 10–30% false-negative results for metastases [12]. Nevertheless, despite serial step section examination of each SLN being possible to overcome the false-negative results, it would be impractical because it requires a heavy workload for pathologists [12]. To overcome this issue, a molecular method, the One Step Nucleic Acid Amplification (OSNA), based on reverse transcription loop mediated isothermal amplification (RT-LAMP) of cytokeratin 19 (CK19) mRNA in the lysate of SLNs, is being adopted worldwide by an increasing number of BC care centers [13]. OSNA has several advantages: allows the analysis of the whole SLN, is semi-quantitative, standardized, reproducible, quicker (30 to 40 min from the excision of the SLN) and also diminish the pathologist workload [12,13,14,15,16,17]. The OSNA cut-off levels were determined by Tsujimoto et al.: macrometastases was defined as >5000 copies/μL of CK19 mRNA, micrometastases as 250 to 5000 copies/μL and a value < 250 copies/μL correspond to absence of metastases or presence of isolated tumor cells (ITC) [14]. Total tumor load (TTL) was defined as the sum of the total number of CK19 mRNA copies in all positive SLNs (in copies/μL) [15,18]. Previous studies revealed that TTL is an independent predictor of the status of the non-sentinel LNs in BC patients and to be independently correlated with disease free survival, local recurrence free survival and overall survival [13,15,18,19,20]. Nevertheless, the exact TTL cut-off to determine ALND is still under debate [13,18,19,21,22]. Moreover, despite all the benefits of the OSNA, one downside of the OSNA assay is the destruction of the SLNs, preventing further microstructural studies that could provide useful information on immune response and tumor aggressiveness.

Although LNs metastases is among the strongest predictors of prognosis, few studies have focused on the assessment of immunoinflammatory response in the LNs and the mechanisms that underlie the local failure of effective anti-tumor immune responses remain poorly understood. In LNs, exposure to tumor-derived factors induces stromal reprogramming, modifies immune cell population dynamics and affects chemokines and interleukins levels, which have the potential to contribute to impaired immune system response [23]. Metastatic LNs are associated with an increased number of plasmacytoid dendritic cells (DCs), regulatory T lymphocytes (Tregs), immature DCs, higher expression of CD163+ M2 macrophages and a lower activation of CD8+ cytotoxic T lymphocytes, suggesting a deficient immune response [1,24]. On the other hand, CD1a DC, mature DC, or CD169+ M1 macrophages are increased in the primary tumor and LNs of patients with non-metastatic LNs, suggesting a more efficient immune response [1,24]. Additionally, on metastatic SLNs, expression of CD83, IL-12p40, IFN-γ, IL-10, and FOXP3 is higher than in non-metastatic SLNs [25]. Thus, prognosis seems to depend not only on whether a patient has LNs metastases or not, but also on the type of local immune-cell population [1,24,26].

Different microenvironments were recognized in each subtype of BC and, consequently, specific microenvironments might be associated with distinct behaviors of the tumor cells and distinct prognosis and potential therapeutic targets [27]. Studies on tumor microenvironment (TME) in patients with Luminal HER2 negative BC are scarce, probably due to the high global survival rates (92.5% at 4 years) and the efficacy of the already existing therapies [28]. Nevertheless, in Luminal HER2 negative BC, 31% had LN metastases and, for this group, the survival rates are lower (84.4% at 4 years) [28]. Considering that about 73% of BC are Luminal HER2 negative, this demonstrates the enormous untapped potential for immuno-targeted therapy in these patients [28,29,30].

As in OSNA assay most of the lysate sample is spared, it can be used for gene expression studies, being the potentially ideal samples to search for new markers related to the immune SLNs response [31,32]. Using a next generation sequencing (NGS) targeted gene expression approach, we aim to identify a transcriptional signature associated with SLNs metastases, in order to identify new biomarkers related to tumor-microenvironment interplay in SLNs of patients with Luminal HER2 negative BC and thereby improve OSNA prognostic information.

## 2. Materials and Methods

### 2.1. Study Design and Participants

This study was an investigator’s initiative, observational, prospective, pilot study. The project was approved by the Ethics Committee of Coimbra Hospital and Universitary Centre (CHUC). Both the project and the informed consent were written according to Good Clinical Practice and the Declaration of Helsinki and the samples were anonymized.

Patients with Luminal A early stage BC (cT1-T2 N0) were invited to participate. The intrinsic subtype classification was based on international guidelines, and it was considered Luminal A BC if ER-positive, HER2-negative, Ki67 < 20% and Progesterone Receptors (PR) ≥ 20% [33,34]. Patients were enrolled until, consecutively, obtain 16 patients with OSNA negative SLNs and 16 with OSNA positive SLNs.

Inclusion criteria were defined including women with invasive BC, Luminal A subtype, cT1-2, cN0, surgical treatment including SLN biopsy and SLN analyzed by OSNA assay. As exclusion criteria, authors defined male BC, age under 18 years-old, pregnancy, germinal mutations associated with breast hereditary cancer, neoadjuvant treatment, cytology proven LN metastases, distant metastases, tumors not expressing CK19, patients unable to give informed consent and technical limitations to SLN biopsy.

### 2.2. SLN Biopsy and OSNA Assay

SLNs were identified under combined techniques, using patent blue and radioisotope or superparamagnetic iron oxide, as previously described [35,36]. After identification by the surgeon, SLNs were removed and directly sent to Pathology Department. The detailed OSNA assay has also been previously described [14,18,37]. In the Pathology Department, after the extra nodal tissue being removed, SLNs that exceeded the specified maximum weight (600 mg) were cut into two or more pieces and processed as separate samples. Then, fresh SLNs were homogenized in 4 mL of a mRNA-stabilizing solution (Lynorhag^®^ solution, Sysmex Corporation, Kobe, Japan) using a RP-10 system (Sysmex Corporation, Kobe, Japan; 90 s at 12,000 rpm). The homogenate (1 mL) was centrifuged for 1 min at 12,200× *g* and the intermediate phase was collected. A volume of 20 µL of the intermediate phase was used for the OSNA assay using the LYNOAMP™ CK19 (Sysmex Corporation, Kobe, Japan) on the RD-210 system (Sysmex Corporation, Kobe, Japan). A standard positive control sample and a negative control sample were used in every assay. Lastly, instead of being discarded, the remaining homogenate was kept at −80 °C for RNA sequencing (RNA-seq) analysis.

The OSNA assay results were based on the calculated number of CK19 mRNA copies/μL, in accordance with the previous cut-off levels: >5000 copies/μL corresponding to macrometastases (pN1), 250 to 5000 copies/μL to micrometastases (pN1mi), and values < 250 copies/μL were classified as negative SLN [14]. In negative SLNs, using RD-210 system (Sysmex Corporation, Kobe, Japan), 160 to 249 corresponded to ITCs [pN0(i+)] and <160 to absence of metastases (pN0) [38]. For each patient, TTL was defined as the sum of CK19 mRNA copies in all positive SLNs [13,18].

The study had two major branches for SLN microenvironment analysis: 16 patients with OSNA positive SLNs and 16 patients with OSNA negative pN0 SLNs. The OSNA positive group was subdivided in SLNs with micrometastases and SLNs with macrometastases. Whenever more than one sample of the SLN or more than one SLN were diagnosed as having metastases, the one with a higher number of copies of CK19 mRNA/μL was considered for gene expression studies.

### 2.3. ALND: Non-Sentinel LNs

The decision to proceed with ALND was discussed in a multidisciplinary team for each patient. Typically, ALND was performed in patients with metastatic SLNs with >15,000 copies/μL of CK19 mRNA or if 3 or more positive SLNs were detected [4,5,18]. Non-sentinel LNs were assessed by current histological and immunohistochemical methods.

### 2.4. Pathological Evaluation of the Tumor

Parameters recorded by the pathologists were tumor’s larger diameter, presence of multicentricity or multifocality, lymphovascular invasion (LVI), histologic type, tumor grade, ER status, PR status, HER2 status, Ki-67 value, molecular classification and tumor infiltrating lymphocytes (TILs) [39,40,41]. Stromal TILs were quantified on hematoxylin and eosin sections of tumor according to the guidelines of the International TILs Working Group [41]. The stromal TILs count was categorized into three grades: low (0–10%), intermediate (10–40%), or high (40–90%) [41,42].

### 2.5. RNA Sequencing

Targeted RNA-seq was performed using the Oncomine™ Immune Response Research Assay—Chef-Ready library preparation kit (Thermo Fisher Scientific, Carlsbad, CA, USA). The Oncomine™ Immune Response Research Assay evaluates the expression of 395 genes, spanning across 36 functional groups, mainly associated with TME interplay (Supplementary Table S1) [43].

For total RNA extraction, OSNA remaining homogenates were centrifuged for 1 min at 16,350 × *g* and 25 µL of the intermediate phase were used for RNA extraction applying the RNeasy^®^ Plus Mini Kit (Qiagen GmbH, Hilden, Germany), according to manufacturer instructions. DNA decontamination of RNA samples were confirmed with two polymerase chain reactions (PCRs). RNA concentrations were determined with the Qubit™ RNA HS Assay Kit and RNA integrity and quality were assessed with Qubit™ RNA IQ Assay, both using Qubit™ 4 Fluorometer (all from Thermo Fisher Scientific, Waltham, MA, USA). Reverse transcription was performed with 10 ng of total RNA using Ion TorrentTM NGS Reverse Transcription Kit (Thermo Fisher Scientific) and a CFX96 thermocycler (Bio-Rad). cDNA (10 µL) was immediately used for automated library preparation with AmpliSeq™ Library Preparation reagents on an Ion Chef™ System (Thermo Fisher Scientific) (20 amplification cycles and 4 min of annealing and extension time). Libraries were quantified by real-time PCR with Ion Library TaqMan™ Quantitation Kit (Thermo Fisher Scientific) in a CFX96 thermocycler (Bio-Rad). Template preparation and chip loading were automatically performed in the Ion Chef™ System using 25 µL of the 50 pM diluted library, Ion 530 Chef Kit and Ion 530 Chip (all from Thermo Fisher Scientific). The sequencing step was performed on the Ion GeneStudio™ S5 Plus System (Thermo Fisher Scientific, Waltham, MA, USA). Base calling and alignment were performed automatically and according to manufacturer’s default pipeline for the Ion GeneStudio™ S5 Plus System (Thermo Fisher Scientific, Waltham, MA, USA). Raw count matrices were obtained using Torrent Suite™ Software 5.16 and Immune Response Torrent Suite™ Plug-in (v5.16.0.0) (Thermo Fisher Scientific, Waltham, MA, USA).

### 2.6. Statistical Analysis

The database is blinded relative to patient identification. The calculations regarding patients, tumors and LNs characteristics were performed with the STATA software, version 13.1. The normal distribution of quantitative variables was evaluated through the Shapiro–Wilk test. Quantitative variables were described with minimum, maximum and mean [± standard deviation (SD)], while categorical variables were described as percentages. Moreover, the following statistical tests were applied when appropriate: Wilcoxon rank-sum test, two sample t-test, Kruskall-Wallis test and one-way ANOVA for continuous variables; Fisher exact test, or chi-square test for categorical variables. Statistical significance was set at *p* < 0.05.

Principal component analysis (PCA) was performed by DESeq2 R package (version 1.36.0), using the complete set of genes, along the first two principal components [44]. Identification of differentially expressed genes (DEGs) for different group comparisons was performed by DESeq2 R package (version 1.36.0), using a false discovery rate (FDR) threshold of 0.05 to correct for multiple hypothesis testing (Benjamini and Hochberg method) [44]. The log2 fold change cut-off for DEGs was set to changes greater than the absolute value of 0.58 (corresponding to a fold change > 1.5 or <0.67). To visualize the differential expression analysis results, a volcano plot was computed using the package EnhancedVolcano (version 1.14.0). A hierarchical clustering heatmap was performed in a data subset, comprising only significant DEGs, using the Pheatmap R package (version 1.0.12). Data was converted using Z-scores and Euclidean distance was used to cluster both genes and samples. Both PCA and hierarquical clustering heatmap used DESeq2 regularized logarithm data transformation (rlog) as input [44]. Subsequent statistical analysis used gene expression data normalized by the median of ratios method using the DESeq2 R package [44]. Two sample t-test, Wilcoxon rank-sum test, one-way ANOVA, Kruskal–Wallis test and Spearman correlation were used to assess the relationship between normalized gene expression and clinical features.

## 3. Results

### 3.1. Clinicopathologic Results

The clinical features of the 32 patients with Luminal A invasive BC included in this study are presented in Table 1, comparing the 16 patients with OSNA negative SLNs (pN0) and the 16 patients with OSNA positive SLNs (pN1 and pN1mi). There were no statistically significant differences related to clinical characteristics (Table 1).

Regarding the histological characteristics of the tumor, the majority were No Special Type (NST) and had a single tumoral focus (Table 2). The OSNA positive group had a higher percentage of LVI, a higher grade and a higher Ki67 (Table 2). There were no statistically significant differences concerning the TILs (Table 2).

The SLNs were identified under combined techniques and the number of removed SLNs were similar in both groups (Table 3). In OSNA positive group, the mean number of metastatic SLNs was 1.1 ± 0.3 (Table 3 and Supplementary Table S2). Micrometastases were found in 43.8% (*n* = 7) and macrometastases in 56.2% (*n* = 9) (Table 3 and Supplementary Table S2). In this study, TTL and OSNA sample result were almost coincident for each patient (rs = 0.997; *p* < 0.001) (Table 3 and Supplementary Table S2). As TTL have a greater and proven clinical relevance, TTL was preferentially considered in subsequent analyses [13,15,19]. In metastatic SLNs, the mean TTL was 121,238.1 ± 213,294.7. In SLNs with micrometastases the mean TTL was 1394.3 ± 1750.0 and in SLNs with macrometastases the mean TTL was 214,450 ± 250,915.2 (*p* < 0.001). ALND was performed in 6 patients (37.5%) and 4 out of 6 patients had metastases in non-sentinel LNs. Among patients submitted to ALND, the mean number of metastatic non-sentinel LNs was 1.7 ± 2.3 (minimum = 0; maximum = 6) and the mean total number of metastatic LNs (sentinel and non-sentinel) was 2.7 ± 0.9 (minimum = 1; maximum = 7). In the group with metastases in non-sentinel LNs (*n* = 4) the mean TTL was 375,975 ± 292,423. Considering the small ALND sample (*n* = 6), there was no statistically significant correlation between the TTL and the number of non-sentinel metastatic LNs (rs = 0.383; *p* = 0.454). However, globally, there was a positive statistically significant correlation with a strong Spearman correlation coefficient between the TTL and the total number of LNs with metastases (sentinel and non-sentinel) (rs = 0.675; *p* = 0.004). Finally, there was also a positive statistically significant correlation between tumor diameter and TTL, with a very strong Spearman correlation coefficient (rs = 0.870; *p* < 0.001). Nevertheless, there were no statistically significant correlations between TTL and other parameters such as age, Body Mass Index (BMI), grade, ER, PR, Ki67, or TILs.

### 3.2. Gene Expression Analysis

We obtained a successful transcript analysis in the 32 cases (100%). Data concerning RNA-seq has been deposited in NCBI’s Gene Expression Omnibus (accession number GSE210006).

**Figure 1:**
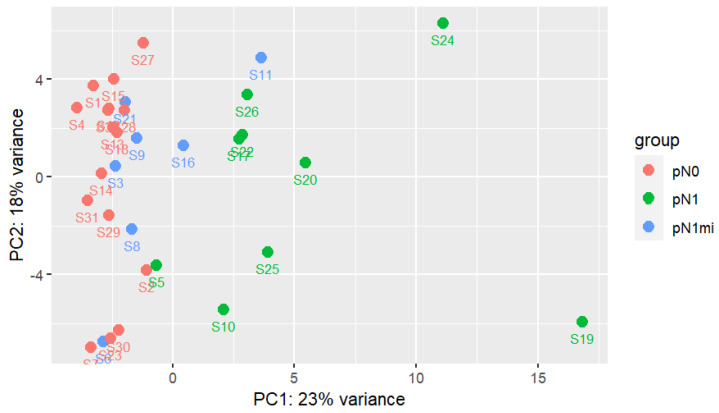
First two principal components analysis with all genes and samples. Each group of samples has a specific color. DESeq2 regularized logarithm data transformation (rlog) was used as input [44].

The result of PCA for the two principal components, including all transcripts and all samples, is shown in Figure 1. The results suggest a high variability and complex pattern of gene expression between samples as the two principal components only explain 41% of samples’ variability. For genes heavily influencing first principal component (PC1) there is higher variation between groups (mainly between pN1 and pN1mi+pN0) than between samples in the same group (as sample-groups are differentiated along PC1). PC1 was unable to segregate samples from pN1mi and pN0 groups. Genes heavily influencing the second principal component (PC2) mainly explain intra group-sample variability, showing a high dispersion. Considering PC1, two pN1 samples stand out, S24 and S19, corresponding both to more severe cases.

**Figure 2:**
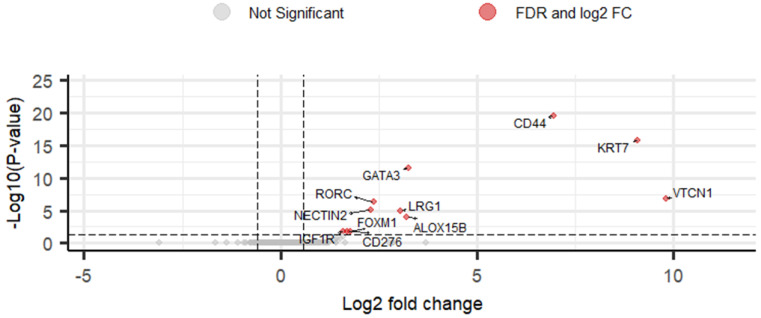
Volcano plot of DESeq2 results for comparison of gene expression levels between pN1 and pN0 using as threshold log2 fold change greater than |0.58| and FDR < 0.05. Eleven differentially expressed genes (DEGs) were identified (red points). Grey points correspond to all other genes. Vertical lines represent the negative and positive values of log2 fold change cut-off. The horizontal line represents the FDR cut-off.

To identify DEGs, DESeq2 R package was used to compare between sample groups (pN0, pN1mi and pN1) [44]. Eleven upregulated DEGs were identified (2.8%) when comparing OSNA positive with macrometastases and OSNA negative SLNs (Figure 2 and Supplementary Table S3). Comparing OSNA positive with micrometastases (pN1mi) and OSNA negative SLNs (pN0), no DEGs were identified. Comparing OSNA positive SLNs with macrometastases and OSNA positive SLNs with micrometastases, 7 DEGs were identified as upregulated (1.8%): *CD44*, *KRT7*, *ALOX15B*, *GATA3*, *LRG1*, *RORC* and *NECTIN2* (Supplementary Table S4). At last, comparing all OSNA positive SLNs (pN1mi and pN1) versus OSNA negative SLNs (pN0), 7 DEGs were identified as upregulated (1.8%): *KRT7*, *VTCN1*, *CD44*, *GATA3*, *ALOX15B*, *RORC* and *NECTIN2* (Supplementary Table S5).

**Figure 3:**
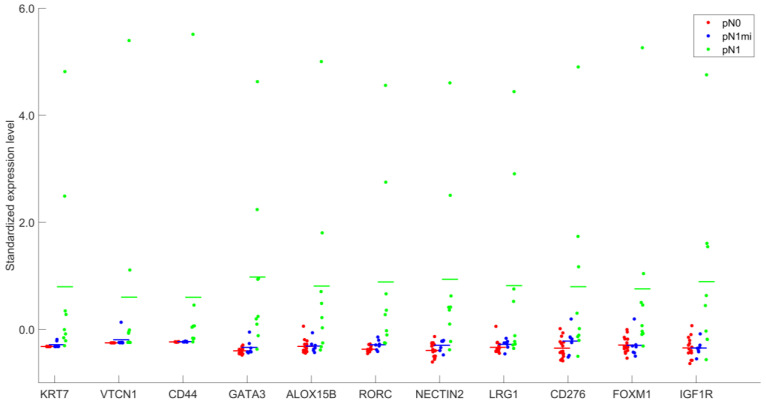
Standardized expression levels after DESeq2 normalization of the 11 genes identified as differentially expressed, for groups pN0, pN1mi and pN1.

Figure 3 and Supplementary Table S6 allow an overview of the levels of expression of the 11 DEGs, identified when comparing OSNA positive SLNs with macrometastases (pN1) and OSNA negative SLNs (pN0).

A gradient of expression from pN0 to pN1 is evidenced, with some genes showing very low levels of expression in non-metastatic SLNs (pN0) (as *KRT7* and *CD44)* and, in particular, completely absent *VTCN1* expression (0.0 ± 0.0) in OSNA negative SLNs (Supplementary Table S6).

### 3.3. Statistical Analysis between DEGs and Relevant Clinicopathologic Parameters

**Figure 4:**
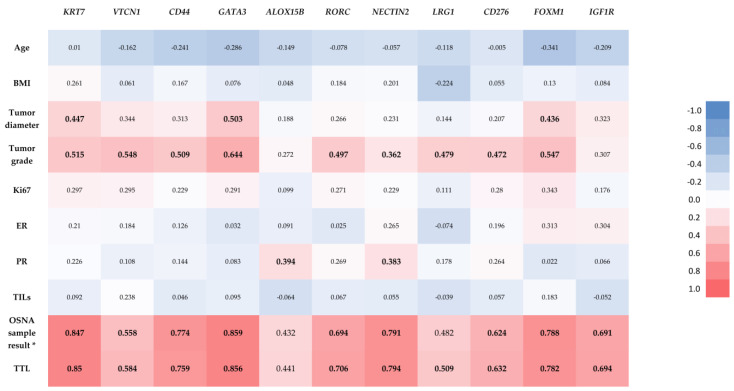
Correlation heatmap with results of Spearman correlation between relevant clinicopathologic parameters and the normalized expression levels of the 11 identified differentially expressed genes. Statistically significant correlations are marked in bold. * CK19 mRNA copies/μL in the selected sample of the OSNA positive samples. The result in OSNA negative samples (<160, non-specified) did not allow Spearman correlation.

The results of Spearman correlation between relevant clinicopathologic parameters and the normalized expression levels of the 11 DEGs (identified from the comparison between pN1 and pN0) are shown in Figure 4.

As shown in Figure 4, Spearman correlation demonstrated a statistically significant positive correlation between TTL and the normalized expression levels of the majority of the DEGs (*KRT7*, *VTCN1*, *CD44*, *GATA3*, *RORC*, *NECTIN2*, *LRG1*, *CD276*, *FOXM1* and *IGF1R*). The OSNA sample result (number of copies of CK19 mRNA/µL) of the analyzed OSNA positive samples also showed statistically significant positive correlations with the same DEGs than TTL, except for *LRG1*. There were also statistically significant positive correlations between the tumor diameter and the normalized expression levels of *KRT7*, *GATA3* and *FOXM1*; between the tumor grade and the normalized expression levels of *KRT7*, *VTCN1*, *CD44*, *GATA3*, *RORC*, *NECTIN2*, *LRG1*, *CD276* and *FOXM1*; and between the PR and the normalized expression levels of *ALOX15B* and *NECTIN2*. There were no statistically significant correlations between the normalized expression levels of any of the 11 identified DEGs and age, BMI, Ki67, ER or TILs.

### 3.4. Clusters

**Figure 5:**
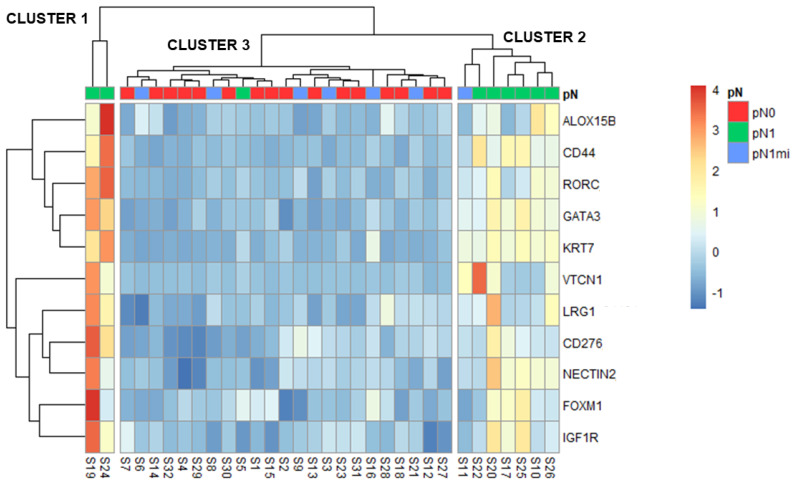
Heatmap and hierarchical clustering with the 11 DEGs (identified when comparing pN1 with pN0). Z-scores are represented by the color scale. Clusters are shown separated.

To identify patterns of gene expression within the 32 samples, a hierarchical clustering heatmap was constructed using the 11 DEGs (identified when comparing pN1 and pN0) using Z-scores (Figure 5).

Three main clusters were identified: cluster 1 including two cases with macrometastases (pN1), cluster 2 with most pN1 cases but also with one micrometastases case (pN1mi) and cluster 3 aggregating pN0, most pN1mi cases and one pN1 (Figure 5). For each cluster, the main characteristics of patients, primary tumors and LNs are compared in Table 4.

The two cases with macrometastases of cluster 1 were from patients that also had non-sentinel LNs with macrometastases (S19 with 6 and S24 with 2) and vessels embolization on the histologic examination of the non-sentinel LNs, though no LVI was described in primary tumors (Table 4). These samples were the two pN1 outliers previously identified in the PCA analysis (Figure 1). Globally, all the 11 DEGs had higher expression in cluster 1 (Figure 5).

Cluster 2 included only OSNA positive cases: 6 with macrometastases and 1 with micrometastases (Figure 5). Importantly, the case with micrometastases that clustered with this group (S11) had a high OSNA sample result and TTL (both 4500) (Supplementary Table S2), close to the established cut-off of macrometastases (5000). Globally, the 11 DEGs were less expressed than in cluster 1 but more expressed than in cluster 3 (Figure 5).

Cluster 3 included all pN0 patients, 6 of the 7 pN1mi patients and one pN1 patient. The mean TTL of the seven OSNA positive patients in this cluster 3 was low (Table 4). The only patient pN1 in this cluster (S5) had the lowest OSNA result and TTL among the patients with macrometastases (both 8200) (Supplementary Table S2). Cluster 3 had the lowest gene expressions of this 11 DEGs (Figure 5).

Finally, besides tumor diameter and tumor grade, the comparison of clinical and other tumor pathological characteristics (hormone receptors, LVI, Ki67 and TILs) between the three clusters did not reveal any statistically significant difference (Table 4).

## 4. Discussion

The cross-talk between immune cells and tumor cells modulates tumor metastases and response to therapy [45]. By binding to inhibitory receptors on immune cells, metastatic cancer cells can disrupt tumor immunity and establish a pro-tumoral microenvironment [45]. Tumors escape immune-mediated recognition through multiple mechanisms [46]. During chronic tumor antigen exposure, T cells become dysfunctional/exhausted and upregulate various checkpoint inhibitory receptors that limit T cell survival and function [46]. In physiological conditions, immune checkpoints (as the identified DEGs *VTCN1* and *CD276*) are crucial to prevent exaggerated inflammation, which would otherwise cause damage to the tissues; however, through upregulation of immune checkpoints, BC cells can also acquire the ability to suppress the immune response and evade recognition and consequent elimination by the immune system [47].

Emerging literature is revealing the potential for the assessment of immune microenvironment in SLNs as predictive biomarkers for treatment by immune checkpoint inhibitors immunotherapies [48]. Indeed, evaluating the immune response within the SLNs could become an easier and more informative measure of therapy efficacy than the assessment of TILs within the primary TME [48]. Previous studies suggested that the presence of primary and metastatic disease promote immune suppression within the SLNs and this may need to be overcome to observe a response to immunotherapy [48].

Gene expression analysis of RNA from SLNs can be used to characterize both cancer and immune cells [49]. Using a targeted RNA-seq, this study revealed DEGs that may be predictive biomarkers in the immune-oncology interface, focusing on the interaction of tumor cells with the microenvironment. When comparing OSNA positive with OSNA negative SLNs, 7 upregulated DEGs were identified, a number that increased to 11, when considering only macrometastatic SLNs (Supplementary Tables S3 and S5). The higher number is probably related to the increased levels of expression, and consequent strengthening statistical power. The upregulated DEGs (*VTCN1*, *KRT7*, *CD44*, *GATA3*, *ALOX15B*, *LRG1*, *RORC*, *NECTIN2*, *CD276*, *FOXM1* and *IGF1R*) (Supplementary Table S3 and Figure 2), include genes mainly expressed in BC cells and genes expressed both in BC and microenvironment cells [according to GeneCards^®^ (RRID: SCR_002773)]. The main function(s) of each DEG, according to Oncomine™ Immune Response Research Assay (Thermo Fisher Scientific, USA) (Supplementary Table S1) and GeneCards^®^ (RRID: SCR_002773), have been previously described: *VTCN1* and *CD276* are inhibitory immune checkpoints; *KRT7* is a tumor marker; *CD44* and *NECTIN2* are mainly correlated with cell-adhesion and migration; *GATA3* is a transcription factor, being required for the T helper cells of type 2 differentiation process; *ALOX15B* regulates cytokine secretion by macrophages; *LRG1* is involved in signal transduction and it is expressed during granulocyte differentiation; *RORC* is a transcription factor with a role in T helper cells of type 17 differentiation; *FOXM1* is also a transcription factor, involved in cell proliferation; *IGF1R* is a growth factor receptor, also involved in cell proliferation. Interestingly, *GATA3* is also one of the most frequently mutated genes in BC and have a strong association with breast tumorigenesis [50]. Remarkably, in our study, genes with an expression that could be mainly attributed to microenvironment cells, as granzymes (*GZMA*, *GZMB*, *GZMH*, *GZMK*), CD3 (*CD3D*, *CD3E*, *CD3G*), other immune system-response related genes codifying interleukins, IFN-γ, T cell receptors (TCRs) or immune checkpoint molecules such as Programmed Cell Death-1 (PD-1) or Cytotoxic T-Lymphocyte Associated Protein 4 (CTLA4), did not show differential expression between pN0, pN1mi and pN1 samples. This lack of evidence of the involvement of other genes associated with immune system activation may possibly be related to the increased expression of the inhibitory immune checkpoints *VTCN1* and *CD276*.

Analysis of the expression levels of the 11 DEGs within the three groups of samples (pN0, pN1mi and pN1), as shown in Figure 3 and Supplementary Table S6, highlights the correlation with the metastatic load of SLNs. Some genes, such as *KRT7*, *VTCN1*, *CD44* or *ALOX15B* had very low or even no expression in pN0 samples (Supplementary Table S6), which is in accordance with the low levels of the respective proteins in LNs, previously described in the literature [51]. On the other hand, concerning metastatic SLNs, increased levels of tumor load were correlated with higher expression levels of the majority of the DEGs (Figure 4), strengthening that the changes in the LNs microenvironment associated with metastases reflect a progressive process. Yet, when evaluating gene expression in OSNA positive samples, the levels of expression cannot be assigned to specific cell types. So, for the identified DEGs, the increased levels of gene expression in the SLNs with higher copies of mRNA CK19/µL may be explained just by tumor load or by the simultaneous overexpression in microenvironment cells in response to metastases. Immunohistochemical studies targeting proteins codified by DEGs would clarify which cells are involved. However, independently of the cell type expressing these biomarkers, they may be useful as prognostic biomarkers and for targeted therapies selection.

Tumor diameter, tumor grade, PR, OSNA sample result and TTL showed positive correlations with DEGs expression levels in SLNs (Figure 4). For tumor diameter and tumor grade, the most probable explanation for these correlations is the association of higher values of these parameters with LN metastases. The weak but statistically significant positive correlations between PR and expression levels of *ALOX15B* and *NECTIN2* in SLNs had not been previously described in the literature. Regarding TTL values and OSNA sample results, as values were almost coincident (Supplementary Table S2), we cannot state if these correlations will stand in patients with a higher number of positive SLNs.

Furthermore, cluster analysis of samples based on the expression profile of the referred 11 DEGs established three different clusters: cluster 1 had the highest gene expression levels whereas cluster 3 had the lowest one (Figure 5). The different clusters, not entirely coincident with pN0, pN1mi and pN1 classification, may relate to distinct prognosis. Based on clinical and pathological characteristics, cluster 1 would have the worst prognosis and cluster 3 the best.

This study is the first in human BC patients analyzing the immune-related DEGs in the whole SLN, comparing the global microenvironment of non-metastatic and metastatic SLNs, and subdividing in micrometastases and macrometastases. A previous study compared the DEGs between metastatic and non-metastatic LN, using a microarray-based dataset (GSE4408) to evaluate 16 metastatic and 3 non-metastatic LNs of mice bearing orthotopic human BC xenografts [52,53]. Rizwan et al., mainly focused on changes in collagen density, investigating extracellular matrix molecules and detected 13 DEGs [52]. Valente et al. compared the DEGs between metastatic and non-metastatic LNs in human BC patients (including all subtypes), however, in metastatic LNs, it was analyzed exclusively the uninvolved (“normal”) residual portion of an otherwise involved LN (using laser microdissection to collect exclusively cell populations of the LN, avoiding the bulk of the tumor and the tumor/LN margins) [54]. The authors used a microarray-based dataset (HG U133A 2.0) to analyze the gene expression and concluded that immune gene expression profiles in uninvolved residual portion of metastatic LNs are significantly different from negative LNs, with 22 DEGs [54]. Blackburn et al., assessing DEGs between non-metastatic LNs in patients with other positive LNs versus non-metastatic LNs in patients with all negative LNs, did not found any DEGs, suggesting that the presence of metastatic cells within the lymphatic system does not elicit widespread changes in gene expression through the remaining LNs; rather, LNs independently respond to disseminated tumor cells [55]. Additionally, Rye et al., in a study using single-cell immune profiling of LNs with and without metastatic cells revealed that immune suppression occurred at early stages of local spread of BC; however, a certain tumor burden must be reached before changes in immune cell distribution can be detected [56]. Thus, the physical presence of metastatic tumor cells may be crucial to elicit a pro-metastatic niche in the LNs and, consequently, the changes in the microenvironment associated with metastases reflect alterations associated with tumor growth and progression [53,54,55,56]. Indeed, in our study, we verify that there are no DEGs between non-metastatic SLN (pN0) and SLN with micrometastases (pN1mi). However, 11 DEGs were identified as upregulated in macrometastatic SLNs (Supplementary Table S3 and Figure 2).

In our study, we used a targeted RNA-seq to study the transcriptomic patterns of immune response at the level of SLNs. Targeted RNA-seq is a very sensitive and specific method, as evidenced by the detection of differential expression of transcripts with very low expression, such as *VTCN1*, supporting the reliability of our results (Supplementary Table S6). Moreover, when compared to the whole transcriptome, beyond reduced costs, targeted RNA-seq protocols are optimized for the selected transcripts, showing increased sensitivity.

Considerable evidence suggests that BC metastases arise from cells undergoing epithelial-mesenchymal transition and cancer stem-like cells. A previous study using single-cell RNA-seq in BC cell lines revealed that migratory BC cells exhibited overall signatures of epithelial-mesenchymal transition and cancer stem-like cells with variable expression of marker genes, and they retained expression profiles of epithelial-mesenchymal transition over time [57]. Indeed, we verify that *CD44* (a molecular marker for cancer stem cells) is overexpressed in metastatic Luminal A BC SLNs, being also a DEG between the macrometastatic and micrometastatic SLNs (Supplementary Tables S3–S5).

The immune response varies widely in metastases across different BC molecular subtypes [45]. In Luminal BC, Núñez et al., observed that Treg frequencies increased with nodal invasion, with a common transcriptomic signature shared by Tregs from tumors and nodes, including CD80 (an immune checkpoint molecule), which is significantly associated with poor patient survival [58]. In our study, by analyzing a broad spectrum of Luminal A BC SLNs (non-metastatic, micrometastatic and macrometastatic, with a wide range of TTL), transcriptomic patterns were revealed, capturing information on the molecular mechanisms and changes in immune composition. Since Luminal A BC patients with metastases in SLNs typically have a higher risk of disease progression and development of distant metastases, these SLNs’ transcriptomic patterns may translate into new therapeutic strategies, including the successful implementation of targeted immunotherapy in Luminal A BC patients.

Although BC was previously considered as a poorly immunogenic cancer, some patients with BC are now expected to benefit from selected immunotherapies [59]. However, the underlying mechanisms of immunotherapy in BC remains incompletely understood and effective clinical biomarkers for BC are still lacking [59]. The identification and optimization of a multiple-biomarker profiling for immunotherapy could help to properly select patients for treatment and to identify rational combination therapies [49,59]. Furthermore, biomarkers may help define the mechanism of action for different agents and help in dose selection and sequencing of drug combinations [49]. To maximize the clinical treatment benefit of cancer immunotherapy, the prediction of the actual immune response by the identification and application of clinically useful biomarkers (therapeutic targets) is required [47]. Indeed, an ideal BC therapeutic target needs to have a crucial role on the biology and/or survival of BC cells, to be highly expressed in BC cells (primary and metastatic) with low heterogeneity and to be expressed in differentiated BC cells as well as in BC initiating cells, because BC cells with low targeted biomarker expression levels tend to generate escape variants under selective pressure [60,61]. Furthermore, the therapeutic target expression on normal tissues must be restricted, preferably at levels below the ones required for effector mechanism activation, in order to minimize toxicity [60,61].

### Strengths and Limitations

As far as we know, this is the first study in human BC patients that intended to analyze the immune-related DEGs in the whole SLN, comparing the global microenvironment of non-metastatic and metastatic SLNs. Furthermore, the metastatic SLNs were classified as micrometastatic and macrometastatic and the microenvironment was compared according to the tumor load.

To the best of our knowledge, this is also the first study in BC patients using a targeted RNA-seq potentially useful for clinical translation. Previous gene expression studies in LNs used mainly microarray-based datasets. In our study, instead of microarrays, we used targeted RNA-seq, a more sensitive and specific method, with a higher quality estimate of protein abundance.

It is known that distinct transcriptomic profiles across molecular subtypes is associated with inter-tumoral heterogeneity of BC [45]. In this study, selecting a homogeneous cohort of patients with Luminal A early stage BC, we established a differential immune transcriptomic profile of Luminal A BC metastatic SLNs and we were able to define three different clusters. Moreover, as the aggressive behavior of BC seems to derive from LNs metastases, these findings could help to take a further step in defining a more precise prognosis of Luminal A BC patients and improving methods of personalized treatments towards higher effectiveness and less side effects. To the best of our knowledge, this study is the first to delineate the immune transcriptomic profile of Luminal A BC SLNs metastases.

Finally, as another major strength, this study is the first to use the OSNA lysate spared sample in the search for biomarkers associated with tumor-microenvironment interplay. The RNA extraction from OSNA lysate spared sample is easier and allows a higher RNA concentration, with higher quality when compared with formalin-fixed paraffin-embedded tumor samples and may constitute an alternative to tumor RNA characterization, particularly when the primary tumor size is small. This approach also has the additional advantage of maintaining the integrity of the primary tumor samples for eventually necessary future studies. Furthermore, since, by law, OSNA lysates samples are, currently, not required to be preserved, OSNA lysates would otherwise be wasted. Therefore, OSNA lysate samples have less ethical and legal implications and, nowadays, have no other utilities besides SLNs staging. Additionally, accordingly to previous DEGs studies, there is a transcriptomic similarity between primary BC and its corresponding LN metastases [53]. Thus, this similarity may translate into new biomarkers using the OSNA lysate spared sample. Lastly, as OSNA is being adopted worldwide by an increasing number of centers in other type of cancers besides BC, RNA-seq in the OSNA lysate spared samples could have a wider utility.

On the other hand, as a limitation, the OSNA lysate samples are obtained from homogenized SLNs, and thus, this approach performs a global evaluation of SLNs’ microenvironment, including tumoral and non-tumoral cells. This limitation is inherent to OSNA sample. RNA-seq deconvolution analysis, a computational method that can simultaneously estimate both sample-specific cell-type proportions and cell-type-specific gene expression profiles using bulk tissue samples, is not feasible in this study because it would require a significantly higher number of targets [62]. However, as already discussed, regardless of the cell of origin, the DEGs can be useful biomarkers.

The overexpression of several potential targets for immunotherapy in metastatic Luminal A BC SLNs represents a promising therapeutic target. However, as this was a RNA-seq study, successful targeting would require further knowledge about the amount and distribution of protein expression, because the RNA-protein correlation may be distorted by posttranscriptional regulation.

Finally, this study had a small sample size and no follow-up data. A larger cohort of patients with subsequent long-term follow-up will be necessary to enrich these results, especially regarding clusters implications.

## 5. Conclusions

Using a targeted RNA-seq, in OSNA remaining lysate of SLNs from Luminal A BC patients, it was found that, in macrometastatic SLNs, there were 11 upregulated genes related to tumor-microenvironment interplay: *KRT7*, *VTCN1*, *CD44*, *GATA3*, *ALOX15B*, *RORC*, *NECTIN2*, *LRG1*, *CD276*, *FOXM1* and *IGF1R.* In metastatic SLNs, higher metastatic load was correlated with higher expression levels of the majority of the DEGs. Hierarchical clustering analysis revealed three different clusters, not completely coincident with pN0, pN1mi and pN1 classification, suggesting that the expression profile of these genes may bring further information on current SLN evaluation.

The 11 identified DEGs codify proteins mainly involved in cancer aggressiveness and with impact in immune response. The overexpression of the immune suppressive genes *VTCN1* and *CD276* may explain that no direct evidence of activation of immune response in metastatic SLNs was found. In the future, the SLN’s microenvironment-related gene-signature study could be used in order to improve prognosis stratification and therapy personalization. As OSNA is being adopted worldwide in another cancers besides BC, RNA-seq in the OSNA lysate could also have utility for other cancer types.

## Figures and Tables

**Table 1:** Clinical characteristics of the study group, comparing patients with OSNA negative SLNs (pN0) and patients with OSNA positive SLNs (pN1 and pN1mi).

Clinical Characteristics	OSNA Negative *N* = 16	OSNA Positive *N* = 16	*p*-Value
**Age, years**			
Minimum	48	43	-
Maximum	78	73	-
Mean ± SD	58.4 ± 7.8	58.1 ± 8.4	0.552 ^¥^
**BMI, kg/m^2^**			
Minimum	18.3	16.6	-
Maximum	33.7	36.3	-
Mean ± SD	25.5 ± 3.5	27.0 ± 4.5	0.148 ^¥^
BMI ≥ 30 kg/m^2^ (%)	6.3% (*n* = 1)	18.8% (*n* = 3)	0.300 ^Φ^
**Gravidity**			
Minimum	0	0	-
Maximum	5	4	-
Mean ± SD	2.1 ± 1.5	2.0 ± 1.1	0.554 ^¥^
**Parity**			
Minimum	0	0	-
Maximum	3	3	-
Mean ± SD	1.8 ± 1.1	1.7 ± 0.9	0.569 ^¥^
**Premenopausal status (%)**	12.5% (*n* = 2)	18.7% (*n* = 3)	0.500 ^Φ^
**Postmenopausal status (%)**	87.5% (*n* = 14)	81.3% (*n* = 13)	
**Age of menopause, years**			
Minimum	45	45	-
Maximum	57	56	-
Mean ± SD	51.5 ±3.7	50.8 ± 3.5	0.705 ^¥^
**Smoker (%)**	18.8% (*n* = 3)	13.2% (*n* = 2)	0.532 ^Φ^

SD—Standard deviation; BMI—Body Mass Index; ¥—Two sample *t*-test; Φ—Fisher’s exact test.

**Table 2:** Histological characteristics of the tumors, comparing patients with OSNA negative SLNs (pN0) and patients with OSNA positive SLNs (pN1 and pN1mi).

Histological Characteristics of the Tumors	OSNA Negative *N* = 16	OSNA Positive *N* = 16	*p*-Value
**Histologic type (%)**			
No special type (NST)	75.0% (*n* = 12)	93.8% (*n* = 15)	0.311 ^Φ^
Lobular	18.8% (*n* = 3)	6.3% (*n* = 1)	
Tubular	6.3% (*n* = 1)	0.0% (*n* = 0)	
**Tumor diameter, mm**			
Minimum	2.0	5.5	-
Maximum	25.0	35.0	-
Mean ± SD	14.1 ± 6.2	16.5 ± 7.6	0.173 ^¥^
**Multifocality or** **multicentricity (%)**	12.5% (*n* = 2)	25.0% (*n* = 4)	0.327 ^Φ^
**LVI (%)**	6.3% (*n* = 1)	43.8% (*n* = 7)	0.019 ^Φ^
**Grade**			
Grade 1 (%)	75.0% (*n* = 12)	31.3% (*n* = 5)	0.020 ^Φ^
Grade 2 (%)	18.8% (*n* = 3)	56.2% (*n* = 9)	
Grade 3 (%)	0.0% (*n* = 0)	12.5% (*n* = 2)	
Unknown	6.3% (*n* = 1)	0.0% (*n* = 0)	
Mean ± SD	1.2 ± 0.4	1.8 ± 0.7	0.006 ^£^
**ER, %**			
Minimum	75	80	-
Maximum	100	100	-
Mean ± SD	91.6 ± 8.7	96.3 ± 6.5	0.081 ^£^
**PR, %**			
Minimum	20	25	-
Maximum	100	100	-
Mean ± SD	63.8 ± 26.8	75.0 ± 21.7	0.101 ^¥^
**Ki67, %**			
Minimum	1	2	-
Maximum	18	18	-
Mean ± SD	7.3 ± 5.1	10.4 ± 4.0	0.034 ^¥^
**TILs, %**			
Low TILs (%)	37.5% (*n* = 6)	31.3% (*n* = 5)	0.878 ^Φ^
Intermediate TILs (%)	31.3% (*n* = 5)	25.0% (*n* = 4)	
High TILs (%)	31.3% (*n* = 5)	37.5% (*n* = 6)	
Unknown	0.0% (*n* = 0)	6.3% (*n* = 1)	
Mean ± SD	29.4 ± 28.6	32.0 ± 17.6	0.647 ^£^

Φ—Fisher’s exact test; SD—Standard deviation; ¥—Two sample t-test; £—Wilcoxon rank-sum (Mann–Whitney) test; LVI—Lymphovascular invasion; TILs—Tumor Infiltrating Lymphocytes.

**Table 3:** Characteristics of the SLNs, comparing patients with OSNA negative SLNs (pN0) and patients with OSNA positive SLNs (pN1mi and pN1).

Characteristics of the SLNs	OSNA Negative *N* = 16	OSNA Positive *N* = 16	*p*-Value
**Technique for SLNs detection (%)**			
Patent blue and radioisotope	56.2% (*n* = 9)	75.0% (*n* = 12)	0.229 ^Φ^
Superparamagnetic iron oxide	43.8% (*n* = 7)	25.0% (*n* = 4)	
**Number of removed SLNs**			
Minimum	1	1	-
Maximum	3	4	-
Mean ± SD	1.7 ± 0.8	1.8 ± 1.0	0.822 ^£^
**Number of metastatic SLNs**			
1 metastatic SLN (%)	-	93.8% (*n* = 15)	-
2 metastatic SLNs (%)	-	6.2% (*n* = 1)	-
Mean ± SD	-	1.1 ± 0.3	-
**OSNA result (%)**			
Negative (pN0)	100% (*n* = 16)	-	-
Micrometastases (pN1mi)	-	43.8% (*n* = 7)	-
Macrometastases (pN1)	-	56.2% (*n* = 9)	-
**OSNA selected sample result**			
Minimum	<160	280	-
Maximum	<160	730,000	-
Mean ± SD	-	118,560 ± 211,763.5	-
**TTL**			
Minimum	-	280	-
Maximum	-	730,000	-
Mean ± SD	-	121,238.1 ± 213,294.7	-

SLN(s)—Sentinel Lymph Node(s); Φ—Fisher’s exact test; SD—Standard deviation; £—Wilcoxon rank-sum (Mann–Whitney) test; TTL—Total Tumor Load, in copies/μL of CK19 mRNA.

**Table 4:** Comparison of the main characteristics between the three identified clusters.

Main Characteristics	Cluster 1 (*N* = 2)	Cluster 2 (*N* = 7)	Cluster 3 (*N* = 23)	*p*-Value
**Age, years** (mean ± SD)	53.0 ± 7.1	56.3 ± 8.4	59.3 ± 7.9	0.443 ^Δ^
**BMI, kg/m^2^** (mean ± SD)	28.6 ± 6.1	27.0 ± 2.3	25.8 ± 4.3	0.555 ^Δ^
**Tumor diameter, mm** (mean ± SD)	25.5 ± 13.4 *^¥^	19.1 ± 5.8	13.2 ± 5.6 *^¥^	0.009 ^Δ^
**Tumor grade** (mean ± SD)	2.5 ± 0.7 *^£^	1.7 ± 0.5	1.4 ± 0.6 *^£^	0.040 ^Ψ^
**ER, %** (mean ± SD)	100.0 ± 0.0	93.6 ± 7.5	93.5 ± 8.3	0.438 ^Ψ^
**PR, %** (mean ± SD)	95.0 ± 7.1	65.7 ± 24.1	68.3 ± 25.1	0.317 ^Δ^
**Ki67, %** (mean ± SD)	8.5 ± 4.9	10.6 ± 4.4	8.3 ± 4.9	0.571 ^Δ^
**Lymphovascular invasion** (%)	0.0% (*n* = 0)	57.1% (*n* = 4)	17.4% (*n* = 4)	0.693 ^Φ^
**TILs, %** (mean ± SD)	20.0 ± 14.1	39.3 ± 25.9	28.9 ± 28.2	0.465 ^Ψ^
**OSNA sample result**				
Minimum	12,000	4500	<160	-
Maximum	730,000	430,000	8200	-
Mean ± SD	371,000 ± 507,702.7	163,071.4 ± 172,381	-	-
**Number of metastatic SLNs** (mean ± SD)	1.0 ± 0.0	1.1 ± 0.4 *^£^	0.3 ± 0.5 **^£^	<0.001 ^Ψ^
**TTL** (mean ± SD)	375,950 ± 500,702.3	167,778.6 ± 176,584.5 *^£^	1922 ± 2974.6 *^£^	0.005 ^Ψ^
**ALND** (%)	100.0% (n = 2)	57.1% (*n* = 4)	0.0% (*n* = 0)	<0.001 ^Φ^
**Number of non-sentinel LNs with metastases** (mean ± SD)	4 ± 2.8	0.5 ± 0.6	-	0.057 ^£^
**Total number of LNs with metastases** (mean ± SD)	5.0 ± 2.8 *^£^	1.4 ± 0.5 *^£^	0.3 ± 0.5 **^£^	<0.001 ^Ψ^
**Total number of LNs with macrometastases** (mean ± SD)	5.0 ± 2.8 *^£^	1.0 ± 0.6 **^£^	0.0 ± 0.2 **^£^	<0.001 ^Ψ^

SD—Standard deviation; BMI—Body Mass Index; ER—estrogen receptor; PR—progesterone receptor; TILs—tumor infiltrating lymphocytes; ^Δ^—One-way ANOVA; * *p* < 0.05; ^¥^—Two sample t-test (between cluster 1 versus cluster 2 + 3; cluster 2 versus cluster 1 + 3; cluster 3 versus cluster 1 + 2, respectively); ** *p* < 0.001; ^£^—Wilcoxon rank-sum (Mann–Whitney) test (between cluster 1 versus cluster 2 + 3; cluster 2 versus cluster 1 + 3; cluster 3 versus cluster 1 + 2, respectively); ^Ψ^—Kruskal–Wallis test; ^Φ^—Fisher’s exact test; TTL—Total Tumor Load, in copies/μL of CK19 mRNA.

## Data Availability

The datasets generated from RNA-seq data and analysed during the current study are available in the NCBI’s Gene Expression Omnibus repository, with the accession number GSE210006.

## Supplementary Materials

The following supporting information can be downloaded at: https://www.mdpi.com/article/10.3390/cancers14235855/s1, Table S1: Gene List—Oncomine Immune Response Research Assay; Table S2: Individualized sample results; Table S3: Genes differentially expressed between OSNA positive SLNs with macrometastases (pN1) and OSNA negative SLNs (pN0); Table S4: Genes differentially expressed between OSNA positive SLNs with macrometastases (pN1) and OSNA positive SLNs with micrometastases (pN1mi); Table S5: Genes differentially expressed between OSNA positive SLNs (pN1 and pN1mi) and OSNA negative SLNs (pN0); Table S6: Normalized expression levels of the 11 genes identified as differentially expressed, comparing pN0, pN1mi and pN1.
