# Supplementary material for: One Step Nucleic Acid Amplification (OSNA) Lysate Samples Are Suitable to Establish a Transcriptional Metastatic Signature in Patients with Early Stage Hormone Receptors-Positive Breast Cancer
Source: Cancers (Basel). 2022 Nov 28;14(23):5855. doi: 10.3390/cancers14235855 (PMC9736102; doi:10.3390/cancers14235855)
Supplement: Supplementary file 1 [file cancers-14-05855-s001.zip › Table S1 - Gene List - Oncomine Immune Response Research Assay.pdf]

Table S1 - Gene List - Oncomine Immune Response Research Assay

Gene	Target	NCBI NAME	NCBI ACCESSION	GENE FUNCTION
**ABCF1**	ABCF1_11741283	ATP_binding_cassette_subfamily_F_member_1	NM_001025091	Housekeeping
**ADGRE5**	ADGRE5_26552764	adhesion_G_protein-coupled_receptor_E5	NM_078481	Adhesion, migration
**ADORA2A**	ADORA2A_771873	adenosine_A2a_receptor	NM_000675	Checkpoint pathway
**AIF1**	AIF1_385491	allograft_inflammatory_factor_1	NM_001623	Macrophage
**AKT1**	AKT1_358467	AKT_serine_threonine_kinase_1	NM_001014431	Tumor marker
**ALOX15B**	ALOX15B_13391445	arachidonate_15-lipoxygenase_type_B	NM_001141	Macrophage
**ARG1**	ARG1_174278	arginase_1	NM_000045	Myeloid marker
**AXL**	AXL_24872596	AXL_receptor_tyrosine_kinase	NM_021913	Innate immune response
**B3GAT1**	B3GAT1_170280	beta-13-glucuronyltransferase_1	NM_018644	NK activation
**BAGE**	BAGE_153290	B_melanoma_antigen	NM_182482	Tumor antigen
**BATF**	BATF_237345	basic_leucine_zipper_ATF-like_transcription_factor	NM_006399	Helper T cells
**BCL2**	BCL2_10401144	B-cell_CLL_lymphoma_2	NM_000633	Apoptosis
**BCL2L11**	BCL2L11_620729	BCL2_like_11	NM_138621	Apoptosis
**BCL6**	BCL6_21502257	B-cell_CLL_lymphoma_6	NM_001706	Type II interferon signaling
**BRCA1**	BRCA1_42344338	BRCA1_DNA_repair_associated	NM_007300	Tumor marker
**BRCA2**	BRCA2_98179922	BRCA2_DNA_repair_associated	NM_000059	Tumor marker

**BST2**	BST2_218322	bone_marrow_stromal_cell_antigen_2	NM_004335	Type I interferon signaling
**BTLA**	BTLA_227334	B_and_T_lymphocyte_associated	NM_181780	Checkpoint pathway
**BUB1**	BUB1_701803	BUB1_mitotic_checkpoint_serine_threonine_kinase	NM_004336	Proliferation
**C10orf54**	C10orf54_562672	chromosome_10_open_reading_frame_54	NM_022153	Checkpoint pathway
**C1QA**	C1QA_67171	complement_component_1_q_subcomponent_A_chain	NM_015991	Innate immune response
**C1QB**	C1QB_111199	complement_component_1_q_subcomponent_B_chain	NM_000491	Innate immune response
**CA4**	CA4_420530	carbonic_anhydrase_4	NM_000717	Neutrophil
**CBLB**	CBLB_296407	Cbl_proto-oncogene_B	NM_170662	T cell receptor signaling
**CCL17**	CCL17_288394	C-C_motif_chemokine_ligand_17	NM_002987	Chemokine signaling
**CCL18**	CCL18_198296	C-C_motif_chemokine_ligand_18	NM_002988	Lymphocyte infiltrate
**CCL2**	CCL2_242346	C-C_motif_chemokine_ligand_2	NM_002982	Lymphocyte infiltrate
**CCL20**	CCL20_297403	C-C_motif_chemokine_ligand_20	NM_004591	Chemokine signaling
**CCL21**	CCL21_432539	C-C_motif_chemokine_ligand_21	NM_002989	Lymphocyte infiltrate
**CCL22**	CCL22_79185	C-C_motif_chemokine_ligand_22	NM_002990	Chemokine signaling
**CCL3**	CCL3_238343	C-C_motif_chemokine_ligand_3	NM_002983	Lymphocyte infiltrate
**CCL4**	CCL4_121220	C-C_motif_chemokine_ligand_4	NM_002984	Lymphocyte infiltrate
**CCL5**	CCL5_110219	C-C_motif_chemokine_ligand_5	NM_002985	Lymphocyte infiltrate
**CCNB2**	CCNB2_9861095	cyclin_B2	NM_004701	Proliferation
**CCR1**	CCR1_54149	C-C_motif_chemokine_receptor_1	NM_001295	Cytokine signaling
**CCR2**	CCR2_385483	C-C_motif_chemokine_receptor_2	NM_001123396	Helper T cells
**CCR4**	CCR4_76182	C-C_motif_chemokine_receptor_4	NM_005508	Chemokine signaling
**CCR5**	CCR5_85193	C-C_motif_chemokine_receptor_5_gene_pseudogene	NM_001100168	Lymphocyte infiltrate
**CCR6**	CCR6_271363	C-C_motif_chemokine_receptor_6	NM_004367	Chemokine signaling
**CCR7**	CCR7_133237	C-C_motif_chemokine_receptor_7	NM_001838	TCR coexpression

**CD14**	CD14_329435	CD14_molecule	NM_000591	Dendridic cell, macrophage
**CD160**	CD160_194300	CD160_molecule	NM_007053	Checkpoint pathway
**CD163**	CD163_21422245	CD163_molecule	NM_004244	Macrophage
**CD19**	CD19_12011317	CD19_molecule	NM_001178098	B cell marker
**CD1C**	CD1C_12531357	CD1c_molecule	NM_001765	Antigen presentation
**CD1D**	CD1D_10661173	CD1d_molecule	NM_001766	Antigen presentation
**CD2**	CD2_350452	CD2_molecule	NM_001767	Lymphocyte infiltrate
**CD209**	CD209_9921101	CD209_molecule	NM_021155	Dendridic cell, macrophage
**CD22**	CD22_10251132	CD22_molecule	NM_001771	B cell marker
**CD226**	CD226_9021006	CD226_molecule	NM_006566	Adhesion, migration
**CD244**	CD244_868973	CD244_molecule	NM_001166663	Checkpoint pathway
**CD247**	CD247_152252	CD247_molecule	NM_198053	TCR coexpression
**CD27**	CD27_409517	CD27_molecule	NM_001242	Drug target
**CD274**	CD274_461569	CD274_molecule	NM_014143	Checkpoint pathway
**CD276**	CD276_12011310	CD276_molecule	NM_001024736	Checkpoint pathway
**CD28**	CD28_601704	CD28_molecule	NM_006139	Checkpoint pathway
**CD33**	CD33_387493	CD33_molecule	NM_001772	Myeloid marker
**CD37**	CD37_496605	CD37_molecule	NM_001774	Lymphocyte infiltrate
**CD38**	CD38_519628	CD38_molecule	NM_001775	Adhesion, migration
**CD3D**	CD3D_400503	CD3d_molecule	NM_000732	TCR coexpression
**CD3E**	CD3E_677780	CD3e_molecule	NM_000733	TCR coexpression

**CD3G**	CD3G_616717	CD3g_molecule	NM_000073	TCR coexpression
**CD4**	CD4_147255	CD4_molecule	NM_000616	Helper T cells
**CD40**	CD40_300407	CD40_molecule	NM_001250	Drug target
**CD40LG**	CD40LG_479579	CD40_ligand	NM_000074	T cell receptor signaling
**CD44**	CD44_10151123	CD44_molecule_Indian_blood_group	NM_000610	Adhesion, migration
**CD47**	CD47_826931	CD47_molecule	NM_001777	Adhesion, migration
**CD48**	CD48_695798	CD48_molecule	NM_001778	Checkpoint pathway
**CD52**	CD52_70175	CD52_molecule	NM_001803	Lymphocyte infiltrate
**CD53**	CD53_28133	CD53_molecule	NM_001040033	Adhesion, migration
**CD6**	CD6_17771886	CD6_molecule	NM_006725	TCR coexpression
**CD63**	CD63_692800	CD63_molecule	NM_001780	Lymphocyte infiltrate
**CD68**	CD68_172280	CD68_molecule	NM_001251	Macrophage
**CD69**	CD69_195303	CD69_molecule	NM_001781	Checkpoint pathway
**CD70**	CD70_259363	CD70_molecule	NM_001252	Drug target
**CD74**	CD74_787896	CD74_molecule	NM_001025159	Antigen processing
**CD79A**	CD79A_184290	CD79a_molecule	NM_001783	B cell receptor signaling
**CD79B**	CD79B_484590	CD79b_molecule	NM_001039933	B cell receptor signaling
**CD80**	CD80_457557	CD80_molecule	NM_005191	Checkpoint pathway
**CD83**	CD83_480580	CD83_molecule	NM_004233	Antigen presentation
**CD86**	CD86_169267	CD86_molecule	NM_175862	Checkpoint pathway
**CD8A**	CD8A_14271531	CD8a_molecule	NM_171827	TCR coexpression
**CD8B**	CD8B_403510	CD8b_molecule	NM_172213	TCR coexpression
**CDK1**	CDK1_837939	cyclin-dependent_kinase_1	NM_001786	Proliferation
**CDKN2A**	CDKN2A_696829	cyclin-dependent_kinase_inhibitor_2A	NM_000077	Tumor marker

**CDKN3**	CDKN3_434534	cyclin-dependent_kinase_inhibitor_3	NM_005192	Proliferation
**CEACAM1**	CEACAM1_10471155	carcinoembryonic_antigen_related_cell_adhesion_molecule_1	NM_001712	Checkpoint pathway
**CEACAM8**	CEACAM8_745847	carcinoembryonic_antigen_related_cell_adhesion_molecule_8	NM_001816	Myeloid marker
**CIITA**	CIITA_294397	class_II_major_histocompatibility_complex_transactivator	NM_000246	Type II interferon signaling
**CLEC4C**	CLEC4C_322429	C-type_lectin_domain_family_4_member_C	NM_130441	Dendridic cell
**CMKLR1**	CMKLR1_36143	chemerin_chemokine-like_receptor_1	NM_001142345	Dendridic cell, macrophage
**CORO1A**	CORO1A_457563	coronin_1A	NM_007074	Lymphocyte infiltrate
**CRTAM**	CRTAM_312417	cytotoxic_and_regulatory_T-cell_molecule	NM_019604	TCR coexpression
**CSF1R**	CSF1R_19612067	colony_stimulating_factor_1_receptor	NM_005211	Cytokine signaling
**CSF2RB**	CSF2RB_11971301	colony_stimulating_factor_2_receptor_beta_common_subunit	NM_000395	Cytokine signaling
**CTAG1B**	CTAG1B_276373	cancer_testis_antigen_1B	NM_001327	Tumor antigen
**CTAG2**	CTAG2_407763	cancer_testis_antigen_2	ENST00000369585	Tumor antigen
**CTLA4**	CTLA4_584689	cytotoxic_T-lymphocyte_associated_protein_4	NM_005214	Drug target
**CTSS**	CTSS_356460	cathepsin_S	NM_004079	Lymphocyte infiltrate
**CX3CL1**	CX3CL1_190298	C-X3-C_motif_chemokine_ligand_1	NM_002996	Type II interferon signaling
**CX3CR1**	CX3CR1_35133	C-X3-C_motif_chemokine_receptor_1	NM_001337	Lymphocyte infiltrate
**CX3CR1**	CX3CR1_147243	C-X3-C_motif_chemokine_receptor_1	NM_001171172	Lymphocyte infiltrate
**CX3CR1**	CX3CR1_207292	C-X3-C_motif_chemokine_receptor_1	NM_001171171	Lymphocyte infiltrate

**CX3CR1**	CX3CR1_58187	C-X3-C_motif_chemokine_receptor_1	NM_001171174	Lymphocyte infiltrate
**CXCL1**	CXCL1_357459	C-X-C_motif_chemokine_ligand_1	NM_001511	Chemokine signaling
**CXCL10**	CXCL10_354459	C-X-C_motif_chemokine_ligand_10	NM_001565	Type II interferon signaling
**CXCL11**	CXCL11_261361	C-X-C_motif_chemokine_ligand_11	NM_005409	Type II interferon signaling
**CXCL13**	CXCL13_202307	C-X-C_motif_chemokine_ligand_13	NM_006419	Type II interferon signaling
**CXCL8**	CXCL8_197302	C-X-C_motif_chemokine_ligand_8	NM_000584	Cytokine signaling
**CXCL9**	CXCL9_149250	C-X-C_motif_chemokine_ligand_9	NM_002416	Type II interferon signaling
**CXCR2**	CXCR2_379486	C-X-C_motif_chemokine_receptor_2	NM_001557	Chemokine signaling
**CXCR3**	CXCR3_11138	C-X-C_motif_chemokine_receptor_3	NM_001504	Chemokine signaling
**CXCR4**	CXCR4_100208	C-X-C_motif_chemokine_receptor_4	NM_003467	Lymphocyte infiltrate
**CXCR5**	CXCR5_153252	C-X-C_motif_chemokine_receptor_5	NM_001716	Type II interferon signaling
**CXCR6**	CXCR6_48153	C-X-C_motif_chemokine_receptor_6	NM_006564	Lymphocyte infiltrate
**CYBB**	CYBB_14221529	cytochrome_b-245_beta_chain	NM_000397	Type II interferon signaling
**DDX58**	DDX58_540643	DEXD_H-box_helicase_58	NM_014314	Interferon signaling
**DGAT2**	DGAT2_439548	diacylglycerol_O-acyltransferase_2	NM_032564	Neutrophil
**DMBT1**	DMBT1_64696575	deleted_in_malignant_brain_tumors_1	NM_007329	Innate immune response
**EBI3**	EBI3_527636	Epstein-Barr_virus_induced_3	NM_005755	T cell regulation
**EFNA4**	EFNA4_548649	ephrin_A4	NM_005227	Tumor marker
**EGFR**	EGFR_31583261	epidermal_growth_factor_receptor	NM_005228	Tumor marker
**EGR2**	EGR2_454559	early_growth_response_2	NM_000399	T cell differentiation
**EGR3**	EGR3_411516	early_growth_response_3	NM_004430	Tumor marker

**EIF2AK2**	EIF2AK2_257357	eukaryotic_translation_initiation_factor_2_alpha_kinase_2	NM_001135651	Type II interferon signaling
**ENTPD1**	ENTPD1_708815	ectonucleoside_triphosphate_diphosphohydrolase_1	NM_001098175	Checkpoint pathway
**EOMES**	EOMES_9391044	eomesodermin	NM_005442	Checkpoint pathway
**FAS**	FAS_473575	Fas_cell_surface_death_receptor	NM_000043	B cell receptor signaling
**FASLG**	FASLG_417521	Fas_ligand	NM_000639	Type II interferon signaling
**FCER1G**	FCER1G_31138	Fc_fragment_of_IgE_receptor_Ig	NM_004106	Lymphocyte infiltrate
**FCGR1A**	FCGR1A_547652	Fc_fragment_of_IgG_receptor_Ia	NM_000566	B cell marker
**FCGR2B**	FCGR2B_792903	Fc_fragment_of_IgG_receptor_IIb	NM_004001	B cell marker
**FCGR3A**	FCGR3A_575682	Fc_fragment_of_IgG_receptor_IIIa	NM_000569	Macrophage
**FCGR3B**	FCGR3B_620722	Fc_fragment_of_IgG_receptor_IIIb	NM_000570	NK activation
**FCRLA**	FCRLA_710815	Fc_receptor_like_A	NM_001184866	B cell marker
**FOXM1**	FOXM1_10551166	forkhead_box_M1	NM_021953	Proliferation
**FOXO1**	FOXO1_9761081	forkhead_box_O1	NM_002015	PD-1 signaling, Tumor marker
**FOXP3**	FOXP3_865975	forkhead_box_P3	NM_014009	T cell regulation
**FUT4**	FUT4_31443252	fucosyltransferase_4	NM_002033	Myeloid marker, stem cell
**FYB**	FYB_12891393	FYN_binding_protein	NM_001465	Lymphocyte infiltrate
**G6PD**	G6PD_10841194	glucose-6-phosphate_dehydrogenase	NM_000402	Housekeeping

**GADD45GIP1**	GADD45GIP1_329429	GADD45G_interacting_protein_1	NM_052850	Apoptosis
**GAGE1,GAGE12I,GAGE12F**	GAGE1_144260	G_antigen_1	NM_001468,NM_001477,NM	Tumor antigen
**GAGE10**	GAGE10_137250	G_antigen_10	NM_001098413	Tumor antigen
**GAGE12J**	GAGE12J_144260	G_antigen_12J	NM_001098406	Tumor antigen
**GAGE13**	GAGE13_116232	G_antigen_13	NM_001098412	Tumor antigen
**GAGE2C,GAGE2A,GAGE2E**	GAGE2C_104217	G_antigen_2C	NM_001472,NM_001127212	Tumor antigen
**GATA3**	GATA3_14061514	GATA_binding_protein_3	NM_001002295	Helper T cells
**GBP1**	GBP1_771872	guanylate_binding_protein_1	NM_002053	Type II interferon signaling
**GNLY**	GNLY_251357	granulysin	NM_006433	NK activation
**GPR18**	GPR18_238340	G_protein-coupled_receptor_18	NM_001098200	TCR coexpression
**GRAP2**	GRAP2_283387	GRB2-related_adaptor_protein_2	NM_004810	TCR coexpression
**GUSB**	GUSB_17671872	glucuronidase_beta	NM_000181	Housekeeping
**GZMA**	GZMA_165265	granzyme_A	NM_006144	Lymphocyte infiltrate
**GZMB**	GZMB_581688	granzyme_B	NM_004131	Lymphocyte infiltrate
**GZMH**	GZMH_233339	granzyme_H	NM_033423	Lymphocyte infiltrate
**GZMK**	GZMK_178286	granzyme_K	NM_002104	Lymphocyte infiltrate
**HAVCR2**	HAVCR2_594705	hepatitis_A_virus_cellular_receptor_2	NM_032782	Checkpoint pathway
**HERC6**	HERC6_17021806	HECT_and_RLD_domain_containing_E3_ubiquitin_protein_ligase_family_member_6	NM_017912	Dendridic cell
**HGF**	HGF_14151518	hepatocyte_growth_factor	NM_000601	Cytokine signaling

**HIF1A**	HIF1A_13371439	hypoxia_inducible_factor_1_alpha_subunit	NM_001530	PD-1 signaling, Tumor marker
**HLA-A**	HLA-A_345445	major_histocompatibility_complex_class_I_A	NM_002116	Antigen processing
**HLA-B**	HLA-B_639746	major_histocompatibility_complex_class_I_B	NM_005514	Antigen processing
**HLA-C**	HLA-C_9191023	major_histocompatibility_complex_class_I_C	NM_002117	Antigen processing
**HLA-DMA**	HLA-DMA_764869	major_histocompatibility_complex_class_II_DM_alpha	NM_006120	Antigen processing
**HLA-DMB**	HLA-DMB_854962	major_histocompatibility_complex_class_II_DM_beta	NM_002118	Antigen processing
**HLA-DOA**	HLA-DOA_55163	major_histocompatibility_complex_class_II_DO_alpha	NM_002119	Antigen processing
**HLA-DOB**	HLA-DOB_182290	major_histocompatibility_complex_class_II_DO_beta	NM_002120	Antigen processing
**HLA-DPA1**	HLA-DPA1_129237	major_histocompatibility_complex_class_II_DP_alpha_1	NM_033554	Antigen processing
**HLA-DPB1**	HLA-DPB1_175280	major_histocompatibility_complex_class_II_DP_beta_1	NM_002121	Antigen processing
**HLA-DQA1**	HLA-DQA1_587686	major_histocompatibility_complex_class_II_DQ_alpha_1	NM_002122	Antigen processing
**HLA-DQA2**	HLA-DQA2_840937	major_histocompatibility_complex_class_II_DQ_alpha_2	NM_020056	Antigen processing
**HLA-DQB2**	HLA-DQB2_142243	major_histocompatibility_complex_class_II_DQ_beta_2	NM_001198858	Antigen processing
**HLA-DRA**	HLA-DRA_186294	major_histocompatibility_complex_class_II_DR_alpha	NM_019111	Antigen processing
**HLA-DRB1**	HLA-DRB1_877982	major_histocompatibility_complex_class_II_DR_beta_1	NM_002124	Antigen processing
**HLA-E**	HLA-E_639747	major_histocompatibility_complex_class_I_E	NM_005516	Antigen processing
**HLA-F**	HLA-F_101204	major_histocompatibility_complex_class_I_F	NM_001098479	Antigen processing

**HLA-F-AS1**	HLA-F-AS1_726834	HLA-F_antisense_RNA_1	NR_026972	Antigen processing
**HLA-G**	HLA-G_483585	major_histocompatibility_complex_class_I_G	NM_002127	Antigen processing
**HMBS**	HMBS_796904	hydroxymethylbilane_synthase	NM_000190	Housekeeping
**ICAM1**	ICAM1_605711	intercellular_adhesion_molecule_1	NM_000201	Type II interferon signaling
**ICOS**	ICOS_382487	inducible_T-cell_costimulator	NM_012092	Checkpoint pathway
**ICOSLG**	ICOSLG_483588	inducible_T-cell_costimulator_ligand	NM_015259	Checkpoint pathway
**ID2**	ID2_579679	inhibitor_of_DNA_binding_2_HLH_protein	NM_002166	T cell regulation
**ID3**	ID3_572678	inhibitor_of_DNA_binding_3_HLH_protein	NM_002167	T cell regulation
**IDO1**	IDO1_268369	indoleamine_23-dioxygenase_1	NM_002164	Drug target
**IDO2**	IDO2_458562	indoleamine_23-dioxygenase_2	NM_194294	Checkpoint pathway
**IFI27**	IFI27_37143	interferon_alpha_inducible_protein_27	NM_005532	Type I interferon signaling
**IFI35**	IFI35_419526	interferon_induced_protein_35	NM_005533	Interferon signaling
**IFI44L**	IFI44L_12771376	interferon_induced_protein_44_like	NM_006820	Interferon signaling
**IFI6**	IFI6_47156	interferon_alpha_inducible_protein_6	NM_022873	Interferon signaling
**IFIH1**	IFIH1_20172123	interferon_induced_with_helicase_C_domain_1	NM_022168	Innate immune response
**IFIT1**	IFIT1_158259	interferon_induced_protein_with_tetratricopeptide_repeats_1	NM_001548	Type I interferon signaling
**IFIT2**	IFIT2_124224	interferon_induced_protein_with_tetratricopeptide_repeats_2	NM_001547	Cytokine signaling
**IFIT3**	IFIT3_72174	interferon_induced_protein_with_tetratricopeptide_repeats_3	NM_001031683	Type I interferon signaling
**IFITM1**	IFITM1_359459	interferon_induced_transmembrane_protein_1	NM_003641	Type I interferon signaling
**IFITM2**	IFITM2_316422	interferon_induced_transmembrane_protein_2	NM_006435	Type I interferon signaling

**IFNA17**	IFNA17_691788	interferon_alpha_17	NM_021268	T cell receptor signaling
**IFNB1**	IFNB1_579686	interferon_beta_1	NM_002176	Type II interferon signaling
**IFNG**	IFNG_400500	interferon_gamma	NM_000619	Type II interferon signaling
**IGF1R**	IGF1R_1229133 8	insulin_like_growth_factor_1_receptor	NM_000875	Adhesion, migration
**IGSF6**	IGSF6_411515	immunoglobulin_superfamily_member_6	NM_005849	Lymphocyte infiltrate
**IKZF1**	IKZF1_189301	IKAROS_family_zinc_finger_1	NM_006060	Lymphocyte development
**IKZF2**	IKZF2_537639	IKAROS_family_zinc_finger_2	NM_016260	Lymphocyte development
**IKZF3**	IKZF3_427534	IKAROS_family_zinc_finger_3	NM_012481	TCR coexpression
**IKZF4**	IKZF4_746852	IKAROS_family_zinc_finger_4	NM_022465	Lymphocyte development
**IL10**	IL10_491598	interleukin_10	NM_000572	Drug target
**IL10RA**	IL10RA_192296	interleukin_10_receptor_subunit_alpha	NM_001558	Lymphocyte infiltrate
**IL12A**	IL12A_739842	interleukin_12A	NM_000882	Drug target
**IL12B**	IL12B_314422	interleukin_12B	NM_002187	Drug target
**IL13**	IL13_268378	interleukin_13	NM_002188	Cytokine signaling
**IL15**	IL15_49155	interleukin_15	NM_000585	T cell regulation
**IL17A**	IL17A_233320	interleukin_17A	NM_002190	Helper T cells
**IL17F**	IL17F_35139	interleukin_17F	NM_052872	Dendridic cell, macrophage
**IL18**	IL18_140246	interleukin_18	NM_001562	T cell regulation
**IL1A**	IL1A_14431546	interleukin_1_alpha	NM_000575	Cytokine signaling
**IL1B**	IL1B_659766	interleukin_1_beta	NM_000576	Type II interferon signaling
**IL2**	IL2_366451	interleukin_2	NM_000586	Drug target

**IL21**	IL21_368450	interleukin_21	NM_021803	Cytokine signaling
**IL22**	IL22_263349	interleukin_22	NM_020525	T cell regulation
**IL23A**	IL23A_386491	interleukin_23_subunit_alpha	NM_016584	Dendridic cell, macrophage
**IL2RA**	IL2RA_240338	interleukin_2_receptor_subunit_alpha	NM_000417	Cytokine signaling
**IL2RB**	IL2RB_9991107	interleukin_2_receptor_subunit_beta	NM_000878	TCR coexpression
**IL2RG**	IL2RG_884988	interleukin_2_receptor_subunit_gamma	NM_000206	Lymphocyte infiltrate
**IL3RA**	IL3RA_465565	interleukin_3_receptor_subunit_alpha	NM_002183	Dendridic cell
**IL4**	IL4_380477	interleukin_4	NM_000589	Cytokine signaling
**IL6**	IL6_521626	interleukin_6	NM_000600	Cytokine signaling
**IL7**	IL7_545652	interleukin_7	NM_000880	Cytokine signaling
**IL7R**	IL7R_551658	interleukin_7_receptor	NM_002185	TCR coexpression
**IRF1**	IRF1_752861	interferon_regulatory_factor_1	NM_002198	Type II interferon signaling
**IRF4**	IRF4_786895	interferon_regulatory_factor_4	NM_002460	Interferon signaling
**IRF9**	IRF9_12191322	interferon_regulatory_factor_9	NM_006084	Type II interferon signaling
**IRS1**	IRS1_37283828	insulin_receptor_substrate_1	NM_005544	Tumor marker
**ISG15**	ISG15_66173	ISG15_ubiquitin-like_modifier	NM_005101	Type I interferon signaling
**ISG20**	ISG20_642750	interferon_stimulated_exonuclease_gene_20	NM_002201	Type I interferon signaling
**ITGA1**	ITGA1_814914	integrin_subunit_alpha_1	NM_181501	Adhesion, migration
**ITGAE**	ITGAE_33753481	integrin_subunit_alpha_E	NM_002208	Adhesion, migration
**ITGAL**	ITGAL_535638	integrin_subunit_alpha_L	NM_002209	Leukocyte migration
**ITGAM**	ITGAM_31033209	integrin_subunit_alpha_M	NM_001145808	Leukocyte migration
**ITGAX**	ITGAX_20482151	integrin_subunit_alpha_X	NM_000887	Dendridic cell

**ITGB1**	ITGB1_679777	integrin_subunit_beta_1	NM_002211	Adhesion, migration
**ITGB2**	ITGB2_15421648	integrin_subunit_beta_2	NM_000211	Lymphocyte infiltrate
**ITGB7**	ITGB7_24342544	integrin_subunit_beta_7	NM_000889	Leukocyte migration
**ITK**	ITK_563669	IL2_inducible_T-cell_kinase	NM_005546	TCR coexpression
**JAML**	JAML_78188	junction_adhesion_molecule_like	NM_001098526	Lymphocyte infiltrate
**JCHAIN**	JCHAIN_134238	joining_chain_of_multimeric_IgA_and_IgM	NM_144646	B cell marker
**KIAA0101**	KIAA0101_319426	KIAA0101	NM_014736	Proliferation
**KIR2DL1**	KIR2DL1_9142	killer_cell_immunoglobulin_like_receptor_two_Ig_domains_and_long_cytoplasmic_tail_1	NM_014218	Drug target
**KIR2DL2**	KIR2DL2_27126	killer_cell_immunoglobulin_like_receptor_two_Ig_domains_and_long_cytoplasmic_tail_2	NM_014218	NK cell marker
**KIR2DL3**	KIR2DL3_654757	killer_cell_immunoglobulin_like_receptor_two_Ig_domains_and_long_cytoplasmic_tail_3	NM_015868	NK cell marker
**KLF2**	KLF2_9111017	Kruppel_like_factor_2	NM_016270	T cell regulation, trafficking
**KLRB1**	KLRB1_177284	killer_cell_lectin_like_receptor_B1	NM_002258	NK activation
**KLRD1**	KLRD1_515619	killer_cell_lectin_like_receptor_D1	NM_002262	Drug target
**KLRF1**	KLRF1_188285	killer_cell_lectin_like_receptor_F1	NM_016523	NK activation
**KLRG1**	KLRG1_410518	killer_cell_lectin_like_receptor_G1	NM_005810	NK activation
**KLRK1**	KLRK1_698804	killer_cell_lectin_like_receptor_K1	NM_007360	NK activation
**KREMEN1**	KREMEN1_12861389	kringle_containing_transmembrane_protein_1	NM_032045	Neutrophil
**KRT5**	KRT5_10631165	keratin_5	NM_000424	Tumor marker
**KRT7**	KRT7_440543	keratin_7	NM_005556	Tumor marker
**LAG3**	LAG3_13111419	lymphocyte_activating_3	NM_002286	Drug target

**LAMP1**	LAMP1_374485	lysosomal_associated_membrane_protein_1	NM_005561	Lymphocyte infiltrate
**LAMP3**	LAMP3_123613<br>44	lysosomal_associated_membrane_protein_3	NM_014398	TCR coexpression
**LAPTM5**	LAPTM5_55566<br>4	lysosomal_protein_transmembrane_5	NM_006762	Lymphocyte infiltrate
**LCK**	LCK_89192	LCK_proto-oncogene_Src_family_tyrosine_kinase	NM_001042771	TCR coexpression
**LCN2**	LCN2_153259	lipocalin_2	NM_005564	Innate immune response
**LEXM**	LEXM_1055115<br>6	lymphocyte_expansion_molecule	NM_001110533	T cell differentiation
**LILRB1**	LILRB1_156816<br>66	leukocyte_immunoglobulin_like_receptor_B1	NM_001081637	Leukocyte inhibition
**LILRB2**	LILRB2_891994	leukocyte_immunoglobulin_like_receptor_B2	NM_001080978	Lymphocyte infiltrate
**LMNA**	LMNA_588696	lamin_A_C	NM_170707	Housekeeping
**LRG1**	LRG1_24131	leucine_rich_alpha-2-glycoprotein_1	NM_052972	Neutrophil
**LRP1**	LRP1_2564267<br>0	LDL_receptor_related_protein_1	NM_002332	Housekeeping
**LST1**	LST1_22120	leukocyte_specific_transcript_1	NM_007161	Leukocyte inhibition
**LY9**	LY9_13751476	lymphocyte_antigen_9	NM_002348	Lymphocyte infiltrate
**LYZ**	LYZ_120222	lysozyme	NM_000239	Innate immune response
**M6PR**	M6PR_229336	mannose-6-phosphate_receptor_cation_dependent	NM_002355	T cell regulation
**MAD2L1**	MAD2L1_1152<br>21	MAD2_mitotic_arrest_deficient-like_1_yeast	NM_002358	Proliferation
**MADCAM1**	MADCAM1_92<br>91024	mucosal_vascular_addressin_cell_adhesion_molecule_1	NM_130760	Adhesion, migration
**MAGEA1**	MAGEA1_2212<br>9	MAGE_family_member_A1	NM_004988	Tumor antigen
**MAGEA10**	MAGEA10_241<br>320	MAGE_family_member_A10	NM_021048	Tumor antigen

**MAGEA12**	MAGEA12_30130	MAGE_family_member_A12	NM_005367	Tumor antigen
**MAGEA3**	MAGEA3_20121	MAGE_family_member_A3	NM_005362,NM_005363	Tumor antigen
**MAGEA4**	MAGEA4_28131	MAGE_family_member_A4	NM_001011548	Tumor antigen
**MAGEC2**	MAGEC2_249358	MAGE_family_member_C2	NM_016249	Tumor antigen
**MAPK1**	MAPK1_13241425	mitogen-activated_protein_kinase_1	NM_002745	Tumor marker
**MAPK14**	MAPK14_14031511	mitogen-activated_protein_kinase_14	NM_139012	Innate immune response
**MELK**	MELK_300401	maternal_embryonic_leucine_zipper_kinase	NM_014791	Proliferation
**MIF**	MIF_157270	macrophage_migration_inhibitory_factor_glycosylation-inhibiting_factor	NM_002415	Innate immune response
**MKI67**	MKI67_581686	marker_of_proliferation_Ki-67	NM_002417	Proliferation
**MLANA**	MLANA_159265	melan-A	NM_005511	Tumor antigen
**MMP2**	MMP2_11051213	matrix_metallopeptidase_2	NM_004530	Tumor marker
**MMP9**	MMP9_11741283	matrix_metallopeptidase_9	NM_004994	Tumor marker
**MPO**	MPO_15121620	myeloperoxidase	NM_000250	Myeloid marker
**MRC1**	MRC1_529634	mannose_receptor_C_type_1	NM_002438	Dendridic cell, macrophage
**MS4A1**	MS4A1_127232	membrane_spanning_4-domains_A1	NM_021950	Drug target
**MTOR**	MTOR_62366338	mechanistic_target_of_rapamycin	NM_004958	PD-1 signaling, Tumor marker
**MX1**	MX1_232336	MX_dynamin_like_GTPase_1	NM_001178046	Interferon signaling
**MYC**	MYC_510619	v-myc_avian_myelocytomatosis_viral_oncogene_homolog	NM_002467	Tumor marker

**NCAM1**	NCAM1_26012709	neural_cell_adhesion_molecule_1	NM_181351	Adhesion, migration
**NCF1**	NCF1_509617	neutrophil_cytosolic_factor_1	NM_000265	Chemokine signaling
**NCR1**	NCR1_754861	natural_cytotoxicity_triggering_receptor_1	NM_004829	NK cell marker
**NCR3**	NCR3_234342	natural_cytotoxicity_triggering_receptor_3	NM_147130	NK cell marker
**NECTIN2**	NECTIN2_13881496	nectin_cell_adhesion_molecule_2	NM_001042724	Adhesion, migration
**NFATC1**	NFATC1_27682875	nuclear_factor_of_activated_T-cells_1	NM_172387	PD-1 signaling
**NFKBIA**	NFKBIA_734840	NFKB_inhibitor_alpha	NM_020529	T cell receptor signaling
**NKG7**	NKG7_557664	natural_killer_cell_granule_protein_7	NM_005601	Lymphocyte infiltrate
**NOS2**	NOS2_91195	nitric_oxide_synthase_2	NM_000625	Innate immune response
**NOTCH3**	NOTCH3_738847	notch_3	NM_000435	Tumor marker
**NRP1**	NRP1_14831588	neuropilin_1	NM_003873	Dendridic cell
**NT5E**	NT5E_12531355	5-nucleotidase_ecto	NM_002526	Checkpoint pathway
**NTN3**	NTN3_12821377	netrin_3	NM_006181	B cell marker
**OAS1**	OAS1_757865	2-5-oligoadenylate_synthetase_1	NM_016816	Type II interferon signaling
**OAS2**	OAS2_15981707	2-5-oligoadenylate_synthetase_2	NM_016817	Interferon signaling
**OAS3**	OAS3_667776	2-5-oligoadenylate_synthetase_3	NM_006187	Interferon signaling
**PDCD1**	PDCD1_143244	programmed_cell_death_1	NM_005018	Drug target
**PDCD1LG2**	PDCD1LG2_315423	programmed_cell_death_1_ligand_2	NM_025239	Checkpoint pathway

**PECAM1**	PECAM1_22132	platelet_and_endothelial_cell_adhesion_molecule_1	NM_000442	Adhesion, migration
**PGF**	PGF_742842	placental_growth_factor	NM_002632	Tumor marker
**PIK3CA**	PIK3CA_12781384	phosphatidylinositol-45-bisphosphate_3-kinase_catalytic_subunit_alpha	NM_006218	PD-1 signaling, Tumor marker
**PIK3CD**	PIK3CD_19842095	phosphatidylinositol-45-bisphosphate_3-kinase_catalytic_subunit_delta	NM_005026	PD-1 signaling, Tumor marker
**PMEL**	PMEL_233339	premelanosome_protein	NM_006928	Drug target
**POLR2A**	POLR2A_42334334	polymerase_RNA_II_subunit_A	NM_000937	Housekeeping
**POU2AF1**	POU2AF1_230337	POU_class_2_associating_factor_1	NM_006235	B cell marker
**PRDM1**	PRDM1_484588	PR_domain_1	NM_001198	PD-1 signaling
**PRF1**	PRF1_672778	perforin_1	NM_005041	NK activation
**PSMB9**	PSMB9_384491	proteasome_subunit_beta_9	NM_002800	Type II interferon signaling
**PTEN**	PTEN_19732078	phosphatase_and_tensin_homolog	NM_000314	PD-1 signaling, Tumor marker
**PTGS2**	PTGS2_14761583	prostaglandin-endoperoxide_synthase_2	NM_000963	Tumor marker
**PTK7**	PTK7_15161625	protein_tyrosine_kinase_7_inactive	NM_002821	Tumor marker
**PTPN11**	PTPN11_14261533	protein_tyrosine_phosphatase_non-receptor_type_11	NM_002834	PD-1 signaling, Tumor marker
**PTPN6**	PTPN6_17741884	protein_tyrosine_phosphatase_non-receptor_type_6	NM_080548	T cell receptor signaling
**PTPN7**	PTPN7_471581	protein_tyrosine_phosphatase_non-receptor_type_7	NM_001199797	Lymphocyte infiltrate
**PTPRC**	PTPRC_710817	protein_tyrosine_phosphatase_receptor_type_C	NM_002838	Lymphocyte infiltrate
**PTPRCAP**	PTPRCAP_383516	protein_tyrosine_phosphatase_receptor_type_C_associated_protein	NM_005608	TCR coexpression

**PVR**	PVR_9571064	poliovirus_receptor	NM_006505	Checkpoint pathway
**PYGL**	PYGL_586697	phosphorylase_glycogen_liver	NM_002863	Neutrophil
**RB1**	RB1_24592560	RB_transcriptional_corepressor_1	NM_000321	Tumor marker
**RORC**	RORC_12011307	RAR_related_orphan_receptor_C	NM_005060	Helper T cells
**RPS6**	RPS6_124229	ribosomal_protein_S6	NM_001010	Tumor marker
**S100A8**	S100A8_239334	S100_calcium_binding_protein_A8	NM_002964	Myeloid marker, MDSC
**S100A9**	S100A9_174280	S100_calcium_binding_protein_A9	NM_002965	Myeloid marker, MDSC
**SAMHD1**	SAMHD1_19002006	SAM_and_HD_domain_containing_deoxynucleoside_triphosphate_triphosphohydrolase_1	NM_015474	Lymphocyte infiltrate
**SDHA**	SDHA_16361735	succinate_dehydrogenase_complex_flavoprotein_subunit_A	NM_004168	Housekeeping
**SELL**	SELL_139245	selectin_L	NM_000655	Leukocyte migration
**SH2D1A**	SH2D1A_540642	SH2_domain_containing_1A	NM_002351	Lymphocyte activation
**SH2D1B**	SH2D1B_173275	SH2_domain_containing_1B	NM_053282	Lymphocyte activation
**SIT1**	SIT1_148254	signaling_threshold_regulating_transmembrane_adaptor_1	NM_014450	Lymphocyte infiltrate
**SKAP2**	SKAP2_809912	src_kinase_associated_phosphoprotein_2	NM_003930	B cell marker
**SLAMF7**	SLAMF7_9161020	SLAM_family_member_7	NM_021181	Drug target
**SLAMF8**	SLAMF8_9071016	SLAM_family_member_8	NM_020125	Lymphocyte infiltrate
**SNAI1**	SNAI1_138246	snail_family_transcriptional_repressor_1	NM_005985	Tumor marker, stemness
**SNAI2**	SNAI2_722828	snail_family_transcriptional_repressor_2	NM_003068	Tumor marker, stemness
**SRGN**	SRGN_230328	serglycin	NM_002727	Lymphocyte infiltrate

**SSX2**	SSX2_233349	SSX_family_member_2	NM_003147	Tumor antigen
**STAT1**	STAT1_18871996	signal_transducer_and_activator_of_transcription_1	NM_007315	Type II interferon signaling
**STAT3**	STAT3_17511856	signal_transducer_and_activator_of_transcription_3	NM_139276	Drug target
**STAT4**	STAT4_245352	signal_transducer_and_activator_of_transcription_4	NM_003151	Helper T cells
**STAT5A**	STAT5A_18501954	signal_transducer_and_activator_of_transcription_5A	NM_003152	Cytokine signaling
**STAT6**	STAT6_231339	signal_transducer_and_activator_of_transcription_6	NM_003153	Helper T cells
**TAGAP**	TAGAP_10531160	T-cell_activation_RhoGTPase_activating_protein	NM_054114	Lymphocyte infiltrate
**TAP1**	TAP1_15701675	transporter_1_ATP-binding_cassette_sub-family_B_MDR_TAP	NM_000593	Type II interferon signaling
**TARP**	TARP_416517	TCR_gamma_alternate_reading_frame_protein	NM_001003806	Lymphocyte infiltrate
**TBP**	TBP_259367	TATA-box_binding_protein	NM_003194	Housekeeping
**TBX21**	TBX21_9251036	T-box_21	NM_013351	Type II interferon signaling
**TCF7**	TCF7_677799	transcription_factor_7_T-cell_specific_HMG-box	NM_003202	Tumor marker
**TDO2**	TDO2_55162	tryptophan_23-dioxygenase	NM_005651	Checkpoint pathway
**TFRC**	TFRC_10891196	transferrin_receptor	NM_001128148	Housekeeping
**TGFB1**	TGFB1_13681474	transforming_growth_factor_beta_1	NM_000660	Checkpoint pathway
**TIGIT**	TIGIT_383491	T-cell_immunoreceptor_with_Ig_and_ITIM_domains	NM_173799	TCR coexpression
**TLR3**	TLR3_24932595	toll_like_receptor_3	NM_003265	Dendridic cell
**TLR7**	TLR7_99206	toll_like_receptor_7	NM_016562	Innate immune response
**TLR8**	TLR8_36137	toll_like_receptor_8	NM_138636	Lymphocyte infiltrate
**TLR9**	TLR9_619727	toll_like_receptor_9	NM_017442	Drug target

**TNF**	TNF_450555	tumor_necrosis_factor	NM_000594	Checkpoint pathway
**TNFAIP8**	TNFAIP8_188295	TNF_alpha_induced_protein_8	NM_014350	Lymphocyte infiltrate
**TNFRSF14**	TNFRSF14_846950	tumor_necrosis_factor_receptor_superfamily_member_14	NM_003820	Checkpoint pathway
**TNFRSF17**	TNFRSF17_254359	tumor_necrosis_factor_receptor_superfamily_member_17	NM_001192	B cell marker
**TNFRSF18**	TNFRSF18_348456	tumor_necrosis_factor_receptor_superfamily_member_18	NM_004195	Drug target
**TNFRSF4**	TNFRSF4_304389	tumor_necrosis_factor_receptor_superfamily_member_4	NM_003327	Drug target
**TNFRSF9**	TNFRSF9_894998	tumor_necrosis_factor_receptor_superfamily_member_9	NM_001561	Drug target
**TNFSF10**	TNFSF10_251357	tumor_necrosis_factor_superfamily_member_10	NM_003810	Apoptosis
**TNFSF13B**	TNFSF13B_774879	tumor_necrosis_factor_superfamily_member_13b	NM_006573	B cell marker
**TNFSF14**	TNFSF14_224334	tumor_necrosis_factor_superfamily_member_14	NM_003807	Checkpoint pathway
**TNFSF18**	TNFSF18_128228	tumor_necrosis_factor_superfamily_member_18	NM_005092	Checkpoint pathway
**TNFSF4**	TNFSF4_347439	tumor_necrosis_factor_superfamily_member_4	NM_003326	Checkpoint pathway
**TNFSF9**	TNFSF9_325412	tumor_necrosis_factor_superfamily_member_9	NM_003811	Cytokine signaling
**TOP2A**	TOP2A_27522855	topoisomerase_DNA_II_alpha	NM_001067	Proliferation
**TP63**	TP63_143246	tumor_protein_p63	NM_003722	Tumor marker
**TRIM29**	TRIM29_882990	tripartite_motif_containing_29	NM_012101	Tumor marker
**TUBB**	TUBB_369479	tubulin_beta_class_I	NM_178014	Housekeeping

**TWIST1**	TWIST1_9291033	twist_family_bHLH_transcription_factor_1	NM_000474	Tumor marker, stemness
**TYROBP**	TYROBP_350455	TYRO_protein_tyrosine_kinase_binding_protein	NM_198125	Lymphocyte infiltrate
**VCAM1**	VCAM1_829933	vascular_cell_adhesion_molecule_1	NM_001078	Leukocyte migration
**VEGFA**	VEGFA_17121817	vascular_endothelial_growth_factor_A	NM_001171623	Chemokine signaling
**VTCN1**	VTCN1_49157	V-set_domain_containing_T_cell_activation_inhibitor_1	NM_024626	Checkpoint pathway
**XAGE1B**	XAGE1B_469547	X_antigen_family_member_1B	NM_001097594	Tumor antigen
**ZAP70**	ZAP70_673781	zeta_chain_of_T_cell_receptor_associated_protein_kinase_70kDa	NM_001079	T cell receptor signaling
**ZBTB46**	ZBTB46_13361440	zinc_finger_and_BTB_domain_containing_46	NM_025224	Dendridic cell
**ZEB1**	ZEB1_74175	zinc_finger_E-box_binding_homeobox_1	NM_001174093	Tumor marker, stemness
