# Supplementary material for: One Step Nucleic Acid Amplification (OSNA) Lysate Samples Are Suitable to Establish a Transcriptional Metastatic Signature in Patients with Early Stage Hormone Receptors-Positive Breast Cancer
Source: Cancers (Basel). 2022 Nov 28;14(23):5855. doi: 10.3390/cancers14235855 (PMC9736102; doi:10.3390/cancers14235855)
Supplement: Supplementary file 1 [file cancers-14-05855-s001.zip › Table S2 - Individualized sample results.pdf]

Table S2 – Individualized sample results

Sample	CK19 mRNA copies/µL in the selected sample	Number of removed SLNs	Number of positive SLNs	TTL	Total number of positive LNs (sentinel and non-sentinel)	TILs (%)	Cluster
S1	<160	3	0		0	50	3
S2	<160	3	0		0	30	3
S3	400	3	1	400	1	5	3
S4	<160	1	0		0	80	3
S5	8 200	1	1	8 200	1	60	3
S6	280	1	1	280	1		3
S7	<160	1	0		0	5	3
S8	620	2	1	620	1	20	3
S9	300	3	1	300	1	5	3
S10	62 000	4	2	62 950	2	40	2
S11	4 500	1	1	4 500	1	80	2
S12	<160	1	0		0	5	3
S13	<160	1	0		0	85	3
s14	<160	3	0		0	20	3
s15	<160	1	0		0	25	3
s16	3 300	1	1	3 300	1	70	3
s17	430 000	1	1	430 000	2	50	2
s18	<160	1	0		0	50	3
s19	730 000	1	1	730 000	7	10	1
s20	290 000	2	1	322 000	2	10	2
s21	360	3	1	360	1	5	3
s22	33 000	1	1	33 000	1	60	2

s23	<160	2	0		0	5	3
s24	12 000	2	1	21 900	3	30	1
s25	300 000	1	1	300 000	1	20	2
s26	22 000	2	1	22 000	1	15	2
s27	<160	1	0		0	15	3
s28	<160	2	0		0	5	3
s29	<160	2	0		0	0	3
s30	<160	2	0		0	15	3
s31	<160	1	0		0	70	3
s32	<160	2	0		0	10	3
