# Supplementary material for: One Step Nucleic Acid Amplification (OSNA) Lysate Samples Are Suitable to Establish a Transcriptional Metastatic Signature in Patients with Early Stage Hormone Receptors-Positive Breast Cancer
Source: Cancers (Basel). 2022 Nov 28;14(23):5855. doi: 10.3390/cancers14235855 (PMC9736102; doi:10.3390/cancers14235855)
Supplement: Supplementary file 1 [file cancers-14-05855-s001.zip › Table S3 - Genes differentially expressed between OSNA positive SLNs with macrometastases (pN1) and OSNA negative SLNs (pN0).pdf]

Table S3. Genes differentially expressed between OSNA positive SLNs with macrometastases (pN1) and OSNA negative SLNs (pN0).

Gene symbol *	Gene name *	Log2 Fold Change	Fold Change	*p*-value	FDR *p*-value
VTCN1	V-set domain containing T cell activation inhibitor 1	9.81	897.6	<0.001	<0.001
KRT7	Keratin 7	9.10	548.7	<0.001	<0.001
CD44	CD44 molecule (Indian blood group)	6.95	123.6	<0.001	<0.001
GATA3	GATA Binding protein 3	3.25	9.5	<0.001	<0.001
ALOX15B	Arachidonate 15-lipoxygenase type B	3.21	9.3	<0.001	<0.001
LRG1	Leucine rich alpha-2-glycoprotein 1	3.06	8.3	<0.001	<0.001
RORC	RAR related orphan receptor C	2.37	5.2	<0.001	<0.001
NECTIN2	Nectin cell adhesion molecule 2	2.29	4.9	<0.001	<0.001
CD276	CD276 molecule	1.78	3.4	<0.001	<0.001
FOXM1	Forkhead box M1	1.71	3.3	<0.001	0.016
IGF1R	Insulin like growth factor 1 receptor	1.60	3.0	<0.001	0.016

*According to HGNC (HUGO Gene Nomenclature Committee).
