# Supplementary material for: One Step Nucleic Acid Amplification (OSNA) Lysate Samples Are Suitable to Establish a Transcriptional Metastatic Signature in Patients with Early Stage Hormone Receptors-Positive Breast Cancer
Source: Cancers (Basel). 2022 Nov 28;14(23):5855. doi: 10.3390/cancers14235855 (PMC9736102; doi:10.3390/cancers14235855)
Supplement: Supplementary file 1 [file cancers-14-05855-s001.zip › Table S4 - Genes differentially expressed between OSNA positive SLNs with macrometastases (pN1) and OSNA positive SLNs with micrometastases (pN1mi).pdf]

**Table S4.** Genes differentially expressed between OSNA positive SLNs with macrometastases (pN1) and OSNA positive SLNs with micrometastases (pN1mi).

| Gene symbol * | Gene name * | Log2 Fold Change | Fold Change | *p*-value | FDR *p*-value |
|---|---|---|---|---|---|
| ***CD44*** | CD44 molecule (Indian blood group) | 6.27 | 77.2 | <0.001 | <0.001 |
| ***KRT7*** | Keratin 7 | 4.99 | 31.8 | <0.001 | 0.012 |
| ***ALOX15B*** | Arachidonate 15-lipoxygenase type B | 3.14 | 8.8 | <0.001 | 0.012 |
| ***GATA3*** | GATA Binding protein 3 | 2.77 | 6.8 | <0.001 | <0.001 |
| ***LRG1*** | Leucine rich alpha-2-glycoprotein 1 | 2.63 | 6.2 | <0.001 | 0.024 |
| ***RORC*** | RAR related orphan receptor C | 2.02 | 4.1 | <0.001 | 0.012 |
| ***NECTIN2*** | Nectin cell adhesion molecule 2 | 1.93 | 3.8 | <0.001 | 0.029 |

*According to HGNC (HUGO Gene Nomenclature Committee).
