# Supplementary material for: One Step Nucleic Acid Amplification (OSNA) Lysate Samples Are Suitable to Establish a Transcriptional Metastatic Signature in Patients with Early Stage Hormone Receptors-Positive Breast Cancer
Source: Cancers (Basel). 2022 Nov 28;14(23):5855. doi: 10.3390/cancers14235855 (PMC9736102; doi:10.3390/cancers14235855)
Supplement: Supplementary file 1 [file cancers-14-05855-s001.zip › Table S5 - Genes differentially expressed between OSNA positive SLNs (pN1 and pN1mi) and OSNA negative SLNs (pN0).pdf]

**Table S5.** Genes differentially expressed between OSNA positive SLNs (pN1 and pN1mi) and OSNA negative SLNs (pN0).

| Gene symbol * | Gene name * | Log2 Fold Change | Fold Change | *p*-value | FDR *p*-value |
|---|---|---|---|---|---|
| ***KRT7*** | Keratin 7 | 8.30 | 315.2 | <0.001 | <0.001 |
| ***VTCN1*** | V-set domain containing T cell activation inhibitor 1 | 7.33 | 160.9 | <0.001 | <0.001 |
| ***CD44*** | CD44 molecule (Indian blood group) | 4.13 | 17.5 | <0.001 | <0.001 |
| ***GATA3*** | GATA Binding protein 3 | 2.58 | 6.0 | <0.001 | <0.001 |
| ***ALOX15B*** | Arachidonate 15-lipoxygenase type B | 2.50 | 5.7 | <0.001 | 0.040 |
| ***RORC*** | RAR related orphan receptor C | 1.79 | 3.5 | <0.001 | 0.025 |
| ***NECTIN2*** | Nectin cell adhesion molecule 2 | 1.72 | 3.3 | <0.001 | 0.042 |

*According to HGNC (HUGO Gene Nomenclature Committee).
