# Supplementary material for: One Step Nucleic Acid Amplification (OSNA) Lysate Samples Are Suitable to Establish a Transcriptional Metastatic Signature in Patients with Early Stage Hormone Receptors-Positive Breast Cancer
Source: Cancers (Basel). 2022 Nov 28;14(23):5855. doi: 10.3390/cancers14235855 (PMC9736102; doi:10.3390/cancers14235855)
Supplement: Supplementary file 1 [file cancers-14-05855-s001.zip › Table S6 - Normalized expression levels of the 11 genes identified as differentially expressed, comparing pN0, pN1mi and pN1.pdf]

Table S6. Normalized expression levels of the 11 genes identified as differentially expressed, comparing pN0, pN1mi and pN1.

	**pN0**<br>N = 16	**pN1mi**<br>N = 7	**pN1**<br>N = 9
KRT7			
Minimum	0.0	0.0	69.5
Maximum	43.5	545.2	20 760.6
Mean ± SD	8.3 ± 12.2	142.6 ± 238.8	4 519.0 ± 7 006.0
VTCN1			
Minimum	0.0	0.0	1.2
Maximum	0.0	71.5	1 050.5
Mean ± SD	0.0 ± 0.0	10.9 ± 26.8	158.7 ± 343.8
CD44			
Minimum	0.0	2.4	8.3
Maximum	14.8	25.4	6 361.5
Mean ± SD	7.4 ± 4.1	12.0 ± 8.4	929.0 ± 2 050.4
GATA3			
Minimum	280.8	425.9	666.2
Maximum	934.9	1 773.2	17 988.7
Mean ± SD	560.4 ± 190.6	783.6 ± 484.6	5 338.1 ± 5 460.9
ALOX15B			
Minimum	0.8	1.8	5.8
Maximum	43.4	33.1	461.5
Mean ± SD	11.6 ± 10.5	12.1 ± 10.3	106.9 ± 144.9
RORC			
Minimum	78.2	92.0	147.4
Maximum	141.9	188.4	1 862.0
Mean ± SD	107.5 ± 17.7	136.6 ± 33.7	554.4 ± 591.3
NECTIN2			
Minimum	48.7	99.5	136.3
Maximum	231.2	203.5	2 049.8
Mean ± SD	131.3 ± 51.7	168.4 ± 41.4	641.4 ± 617.0
LRG1			
Minimum	20.6	15.8	61.2
Maximum	245.7	145.8	2 210.4
Mean ± SD	70.3 ± 54.5	95.0 ± 41.4	586.9 ± 765.4
CD276			
Minimum	35.6	46.5	48.7
Maximum	127.7	155.2	875.7
Mean ± SD	72.0 ± 28.2	92.1 ± 36.6	247.6 ± 260.1
FOXM1			
Minimum	52.5	61.7	107.5
Maximum	182.5	230.7	1 463.6
Mean ± SD	112.2 ± 35.9	110.7 ± 56.2	367.5 ± 423.1
IGF1R			
Minimum	645.2	823.8	794.1
Maximum	2 090.1	1 777.4	11 664.7
Mean ± SD	1 241.1 ± 382.5	1 237.7 ± 290.7	3 768.6 ± 3 344.0

SD –Standard deviation.
